# The Race against Protease Activation Defines the Role of ESCRTs in HIV Budding

**DOI:** 10.1371/journal.ppat.1005657

**Published:** 2016-06-09

**Authors:** Mourad Bendjennat, Saveez Saffarian

**Affiliations:** 1 Department of Physics and Astronomy, University of Utah, Salt Lake City, Utah, United States of America; 2 Center for Cell and Genome Science, University of Utah, Salt Lake City, Utah, United States of America; 3 Department of Biology, University of Utah, Salt Lake City, Utah, United States of America; Vanderbilt University School of Medicine, UNITED STATES

## Abstract

HIV virions assemble on the plasma membrane and bud out of infected cells using interactions with endosomal sorting complexes required for transport (ESCRTs). HIV protease activation is essential for maturation and infectivity of progeny virions, however, the precise timing of protease activation and its relationship to budding has not been well defined. We show that compromised interactions with ESCRTs result in delayed budding of virions from host cells. Specifically, we show that Gag mutants with compromised interactions with ALIX and Tsg101, two early ESCRT factors, have an average budding delay of ~75 minutes and ~10 hours, respectively. Virions with inactive proteases incorporated the full Gag-Pol and had ~60 minutes delay in budding. We demonstrate that during budding delay, activated proteases release critical HIV enzymes back to host cytosol leading to production of non-infectious progeny virions. To explain the molecular mechanism of the observed budding delay, we modulated the Pol size artificially and show that virion release delays are size-dependent and also show size-dependency in requirements for Tsg101 and ALIX. We highlight the sensitivity of HIV to budding “on-time” and suggest that budding delay is a potent mechanism for inhibition of infectious retroviral release.

## Author Summary

ESCRTs are implicated in cellular processes which require fission of budding membranes. Likely the most studied of these processes is the HIV-ESCRT interactions. The canonical view is that interference with ESCRT recruitment results in a late budding arrest of virions at the plasma membrane and this mechanistic view of ESCRTs has shaped our understanding of their function in almost all cell biology. In this manuscript, we present a full kinetic analysis of HIV virion release under all known mutations in Gag that affect HIV-ESCRT interactions. Our data show that contrary to the canonical view, a defect in ESCRT recruitment does not inhibit virion budding, however it creates a delay. We further show that during budding delay, activated proteases release critical HIV enzymes back to host cytosol, leading to budding of non-infectious progeny virions. We suggest that budding delay is a potent mechanism for inhibition of infectious retroviral release and can be the basis for developing antiviral treatments which slow the budding process and therefore disproportionally affect infectious retroviral release. We also suggest that such budding delay may be one of the mechanisms underlying cellular innate immune responses which inhibit the spread of retroviral infection.

## Introduction

HIV incorporates an aspartic protease that requires homo-dimerization for activation and is the target of numerous FDA approved inhibitors [1–3]. The monomeric form is encoded within the immature virion as part of the Gag-Pol precursor which includes Matrix (MA), Capsid (CA), Spacer Peptide 1 (SP1), Nucleocapsid (NC), Transframe (TF), Protease (PR), Reverse Transcriptase (RT), and Integrase (IN) domains [4]. There are ~120 Gag-Pol proteins packaged in each immature HIV virion along with ~2,000 Gag proteins. Gag and Gag-Pol are synthesized from the same messenger RNA via a ribosomal slippage, therefore Gag has the same N terminal sequence as Gag-Pol with MA, CA, SP1, NC, plus the Gag-specific Spacer Peptide 2 (SP2) and the unstructured p6 domain that is essential for budding of infectious virions [5–7]. Protease activation is vital for auto-processing of Gag-Pol, which in turn is essential for maturation and infectivity of HIV virions [8,9]. The protease activity within Gag-Pol is highly regulated and the release from its boundaries in Gag-Pol, especially the TF domain, substantially increases its activity [10–12]. There are eleven canonical protease sites on Gag and Gag-Pol precursors, and *in vitro* experiments using recombinant PR and HIV Gag as substrate, have characterized the affinities of PR to these sites (from high to low affinity: SP1/NC, SP2/p6, MA/CA, NC/SP2 and CA/SP1 sites) [4,13,14]. Once Gag is processed, the newly released CA assembles within the virion cavity to form the HIV mature capsid which encapsidates the RNA bound to Gag NC along with RT and integrase [7]. While the HIV protease has been studied extensively, the mechanism and timing of its initial activation *in vivo* has remained elusive, and the putative connection between protease activation and the endosomal sorting complexes required for transport (ESCRTs), which support HIV budding [15], remains unexplored.

ESCRTs are implicated in cellular processes which require fission of budding membranes and are shown to play a major role in multivesicular body formation [16], enveloped virus budding [15], cytokinesis [17–19], exosomal vesicle generation [20], and plasma membrane repair [21]. Likely, the most studied of these processes is the impact of ESCRTs on HIV budding. The unstructured p6 domain of Gag hosts two major ESCRT interaction motifs, PTAP and YP [22–24]. The PTAP motif directly interacts with Tsg101 [25–28], and its mutation has a severe effect on HIV virion infectivity. The YP motif interacts with ALIX [29–33]; ALIX also interacts with the upstream Gag NC domain however the exact function of this interaction is still not fully clear [34,35]. The PTAP and YP motifs are collectively known as HIV late domains; indeed, many enveloped viruses interact with early ESCRTs through specific domains within their matrix protein termed late domains [15]. The late domain terminology stems from the observed phenotype of late budding arrest visualized by electron microscopy of budding viruses with altered late domains [5,15,27,36,37]

Here we found that late budding arrest of HIV, due to mutations of its late domains, is transient. We have characterized the budding kinetics starting with Gag virus like particles (VLPs). HIV Gag protein is sufficient for assembly of Gag VLPs with the same size as HIV virions [38]. Using Gag VLPs, we show that Gag with mutated PTAP (ΔPTAP) or YP (ΔYP) motif releases out of plasma membrane with ~1 hour and ~20 minutes delay compared to WT, respectively. To analyze the effect of the same mutations on VLPs incorporating both Gag and Gag-Pol, we generated a fully functional Gag.Pol vector that incorporates both Gag and Gag-Pol and is sufficient for budding mature VLPs with similar efficiency to HIV-1 full-length virus. We show that with an active protease, the Gag.Pol VLP budding is delayed when introducing ΔPTAP and ΔYP mutations. Indeed, Gag.Pol VLPs with ΔPTAP mutation are released with ~10 hours delay and are void of HIV RT and PR. Gag.Pol VLPs with ΔYP mutation are released with ~75 minutes delay, which results in significant reduction of RT and PR incorporation within released VLPs. Budding of Gag.Pol VLPs with an inactive protease and either ΔPTAP or ΔYP mutations is dramatically slowed down with similar sensitivity to the involvement of Tsg101 and ALIX. Using Gag proteins with multiple GFP fusions as cargo, we further show that budding is sensitive to the size of cargo proteins, and this effect is reproduced when a PR inactive truncated Pol protein is used as cargo. Finally, modeling these data using *MonteCarlo* simulations show that the protease activation after complete assembly of HIV virions on plasma membrane can quantifiably explain the loss of Pol specific proteins to host cell cytosol before VLP release.

## Results

Humanized HIV Gag protein expressed in cells supports production of VLPs with similar size distributions as HIV virions [38]. We initiated our study by performing side by side comparison between budding of HIV Gag VLPs versus VLPs produced from the HIV-1 ΔR8.2 vector (HIV_R8.2_) and its parental full length HIV-1 R9 vector (HIV_R9_). HIV_R8.2_ after budding incorporates all components of the virion except ENV proteins and the genomic RNA.

### In contrast to HIV_R9_ and HIV_R8.2_ budding, Gag VLP release is not sensitive to p6 alterations

**Fig 1:**
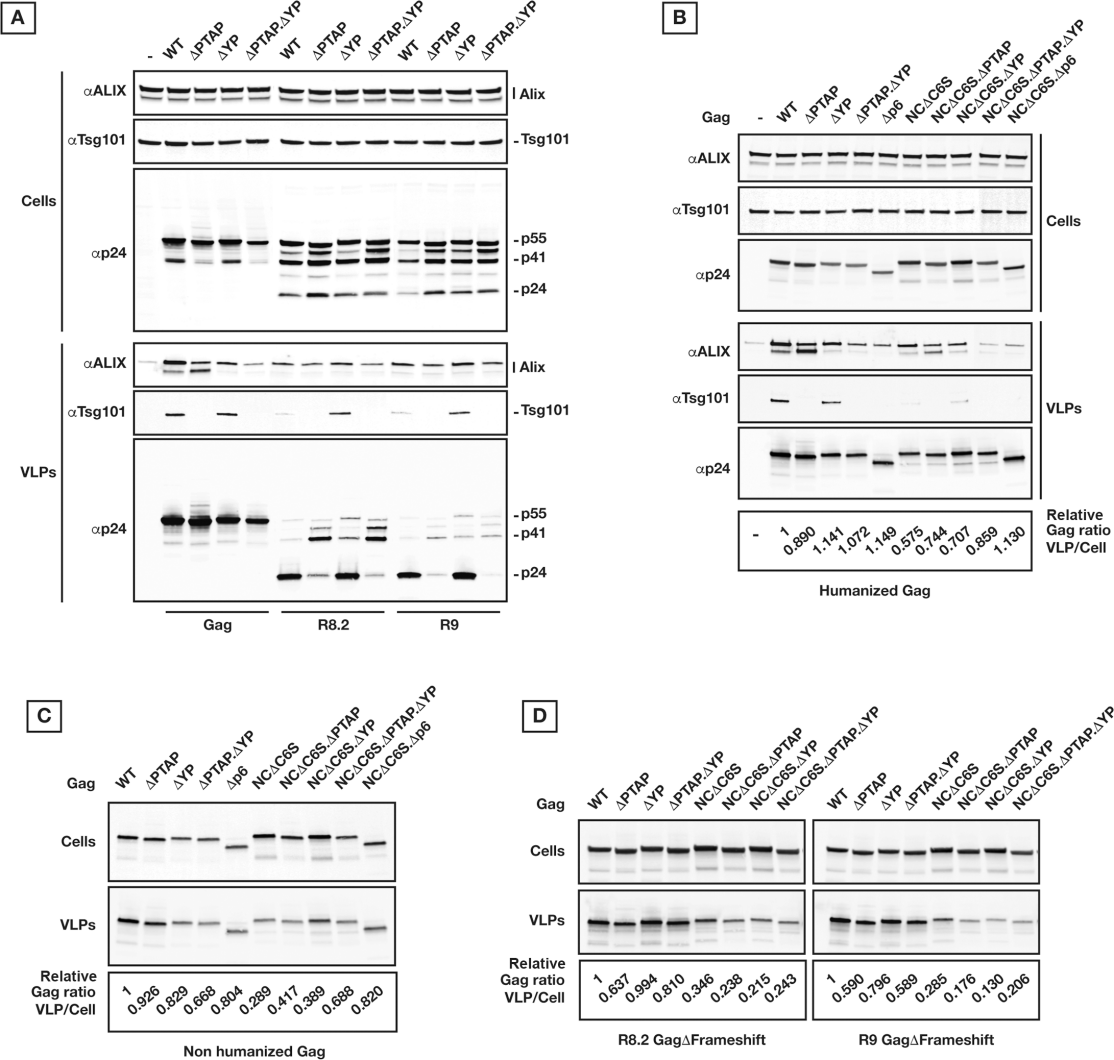
VLP release by Gag and HIV are differently sensitive to PTAP and YP inactivation. **(A)** Gag versus HIV_R9_ and HIV_R8.2_ expressions in 293T cells and corresponding virion/VLP release. 200 ng of each construct was used for transfection, and samples were collected 24 hours post-transfection. Mutations in primary binding sites of Tsg101 and ALIX (PTAP and YP, respectively) and the subsequent retention of Tsg101 and ALIX in the released VLPs are shown. **(B)** Effect of Gag mutations on the yield of VLP release in 293T cells. 200 ng of each humanized Gag construct were used for transfection, and samples were collected 24 hours post-transfection. Tsg101 and ALIX retention in VLPs is also shown. **(C)** Effect of Gag mutations on the yield of VLP release in 293T cells by non-humanized Gag. 1 μg of each Gag construct plus 300 ng HIV Rev encoding vector were used, and samples were collected 24 hours post-transfection. **(D)** Effect of Gag mutations on the yield of VLP release in 293T cells by non-humanized Gag in the context of HIV. The ribosomal slippage site on Gag cDNA was inactivated by mutagenesis without changing the corresponding translated amino acids. 250 ng of each GagΔFrameshift construct were used and samples were collected 24 hours post-transfection. Densitometry values correspond to the ratio of p24 in VLPs/Cells relative to WT values. All experiments were performed at least 3 times with similar results; specifically, the variability in the final densitometry values is <0.05).

In parallel experiments, budding of HIV_R9_ and HIV_R8.2_ were compared to Gag VLP release 24 hours after transfection. Using Gag domain mutagenesis, we observed that while HIV release and maturation are affected by p6 late domain mutations, VLP production by Gag remains almost unaffected (**Fig 1A**). Shown are the following mutations in Gag p6, HIV_R9_ and HIV_R8.2_: ΔPTAP incorporate a _7_LIRL_10_ instead of _7_PTAP_10_ [6], ΔYP include _36_SR_37_ instead of _36_YP_37_ [31], and ΔPTAP. ΔYP has both PTAP and YP sequences altered (_7_LIRL_10_ plus _36_SR_37_). 24 hours post-transfection, cells and VLPs were collected as described in Materials and Methods and analyzed by immunoblotting using p24, ALIX and Tsg101 specific antibodies.

We found that incorporation of early ESCRTs in released Gag as well as HIV_R9_ and HIV_R8.2_ VLPs was sensitive to late domain mutagenesis; Tsg101 was fully sensitive to ΔPTAP mutation, and ALIX was only slightly affected by ΔYP mutation (**Fig 1A**). ALIX migrates as two separate bands with the upper band likely related to a post-translational modified form. We don’t know yet at this stage the nature of this ALIX modification. ALIX background level corresponds to exosome release **(Fig 1A and 1B)**. As commonly reported, HIV virion release (here shown HIV_R9_ and HIV_R8.2_) was detectably reduced under the ΔPTAP mutation in addition to a clear defect in Gag processing. Also, a slight change in release and maturation profiles was observed in ΔYP mutant HIV_R9_ and HIV_R8.2_.

In contrast to HIV_R9_ and HIV_R8.2_, production of Gag VLPs was only slightly affected by mutations within p6 (**Fig 1**). Indeed, expression of Gag with alteration in late domains either as humanized, non-humanized co-expressed with Rev or within R9 with an abrogated ribosomal slippage leads to the same results (**Fig 1B, 1C and 1D**). Aside from interacting with the p6 domain, ALIX also binds to the NC domain and mutations affecting NC have recently been implicated in HIV virion release [39]. We found that under NCΔC6S (replacement of each NC cysteine by serine) plus ΔYP and ΔPTAP mutations, ALIX retention in released Gag VLPs is further abrogated and a reduction in TSG101 retention was observed in Gag NCΔC6S VLPs (**Fig 1B**), however, none of the amino acid substitutions and/or truncations had a marked effect on Gag VLP release (see also **S1A**, **S1B** and **S1C Fig**). The observations related to Gag versus HIV_R8.2_ VLP release were confirmed by pulse/chase ^35^S-labeling experiments (**S2A Fig**).

The Tsg101/ALIX engagement-independent release of Gag VLPs was tested on different cell types with no major changes in the VLP release except for the apparent cell type specific NC effect (**S3 Fig**). Plasma membrane binding requirement was tested by G2A mutation [40] which abrogated VLP budding (**S1A Fig**).

### CHMP4 and VPS4 are retained in released Gag VLPs in absence of functional p6 late domains and VPS4 is essential for release of Gag VLPs

**Fig 2:**
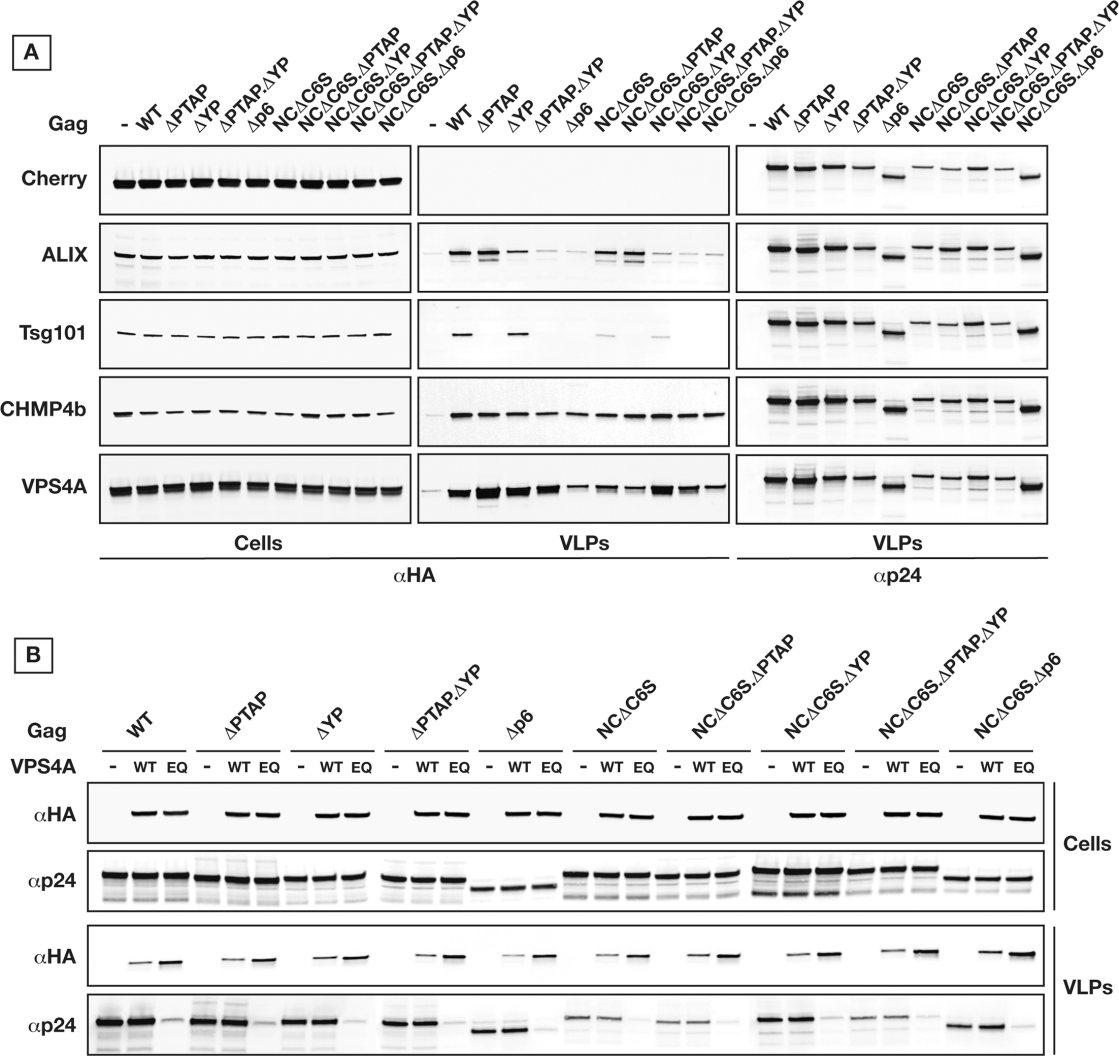
Gag VLP release can bypass ESCRT-I/ALIX for recruitment of ESCRT-III/VPS4. **(A)** ESCRT-III/VPS4 is retained within released VLPs independently of ESCRT-I/ALIX recruitment. The ESCRT proteins, Tsg101, ALIX, CHMP4b, and VPS4A, were co-expressed as HA-tagged ORFs along with the Gag variants in 293T cells as indicated. Their retention in released VLPs indicates their recruitment during VLP budding. **(B)** Expression of dominant negative VPS4 inhibits VLP release by Gag. 293T cells were transfected two times successively at 24 hours interval with HA tagged VPS4 either wild type (WT) or dominant negative E228Q (EQ) then with the Gag variants as indicated. Cells and VLPs were collected 24 hours post-Gag transfection. All experiments were performed 3 times with similar results.

Given that compared to HIV_R9_ and HIV_R8.2_, Gag VLP production 24 hours post-transfection shows differential dependence on late domains, we set out to test the requirements of higher ESCRT factors in release of Gag VLPs. To investigate ESCRT recruitment (Tsg101, ALIX, CHMP4b, and VPS4A) to budding Gag VLPs, we used HA-tagged forms under tight control of their expression levels along with Gag NC and/or p6 mutants (**Fig 2A**). We found that Gag VLPs were released with similar yield even when recruitment of Tsg101 and ALIX were compromised due to p6 and/or NC mutations, however surprisingly these VLPs retained both CHMP4b and VPS4A independently of p6 and NC alterations that are inhibiting the early ESCRTs recruitments. Fluorescently tagged ESCRT-III components have been previously localized within budding wild type Gag VLPs, however not in the presence of p6 mutations [41]. Even if our observation is based on a mild over-expression, it clearly shows that ESCRT-III and VPS4 have the potential to be recruited independently of ESCRT-I/ALIX.

We further tested the requirement for VPS4 engagement in production of VLPs with compromised interactions with early ESCRTs. To this end, we expressed a dominant negative VPS4 (ΔE228Q) during production of HIV Gag VLPs. As shown in **Fig 2B**, the expression of VPS4ΔE228Q had a substantial negative effect on all Gag VLP production which confirms a requirement for VPS4 in eventual Gag VLP production.

### Gag VLP release is delayed when p6 domains are altered

**Fig 3:**
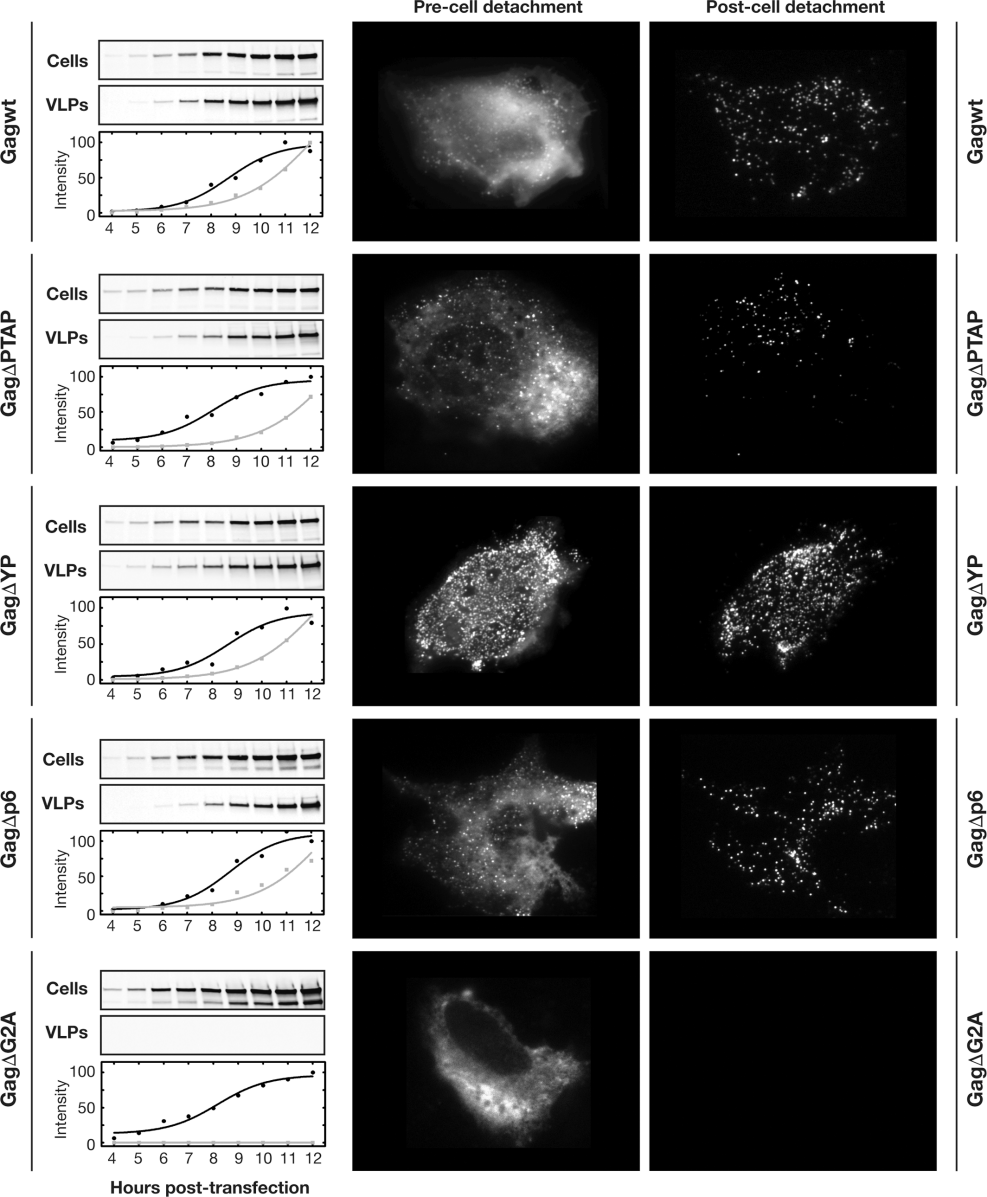
Gag p6 alteration delays Gag VLP release. Kinetics of VLP release by Gag in U2OS cells with either p6 wild type or inactivated as indicated, western blot kinetics are shown where 200 ng of each Gag construct was used for transfection; both Cells and VLPs were collected at 1 hour intervals and immunoprobed using p24 antibody (left panels), Single cell imaging 12 hours post-transfection of mCherry fused Gag constructs as indicated was performed using TIRF microscopy, images were captured before and after cell detachment to visualize released VLPs (right panels). All experiments were performed 3 times with similar results.

While our data show that Gag VLPs with compromised interactions with Tsg101 and ALIX were released with similar efficiencies 24 hours post-transfection, we further investigated the effect of these interactions on the kinetics of Gag VLP production. Shown in **Fig 3** is the VLP production comparing WT, ΔPTAP, ΔYP, Δp6 and control ΔG2A. U2OS cells were used for both immunoblotting and microscopy (left and right panels, respectively). Our analysis shows that the kinetics of VLP release is delayed by ~20 minutes (ΔYP) to ~1 hour (ΔPTAP and Δp6) which is consistent with the similar VLP release observed 24 hours post-transfection (see also Supporting Information section).

To confirm that the Gag variants detected in VLPs using immunoprobing indeed originate from VLPs produced by cells, we visualized the released VLPs by total internal reflection microscopy (TIRF) on individual cells. Using TIRF and Gag p6 variants fused to mCherry, we followed the assembly and release of VLPs in live cells. We confirmed the similar VLP assembly on cellular plasma membrane between all Gag variants, and 12 hours post-transfection, we artificially detached the cells to visualize released VLPs as described in Materials and Methods. VLPs were indeed observed immobilized on the cell-free surface accordingly as shown in **Fig 3** (left panels).

### Humanized Gag-Pol vector preserving the ribosomal slippage produces VLPs that mature similarly to HIV VLPs

**Fig 4:**
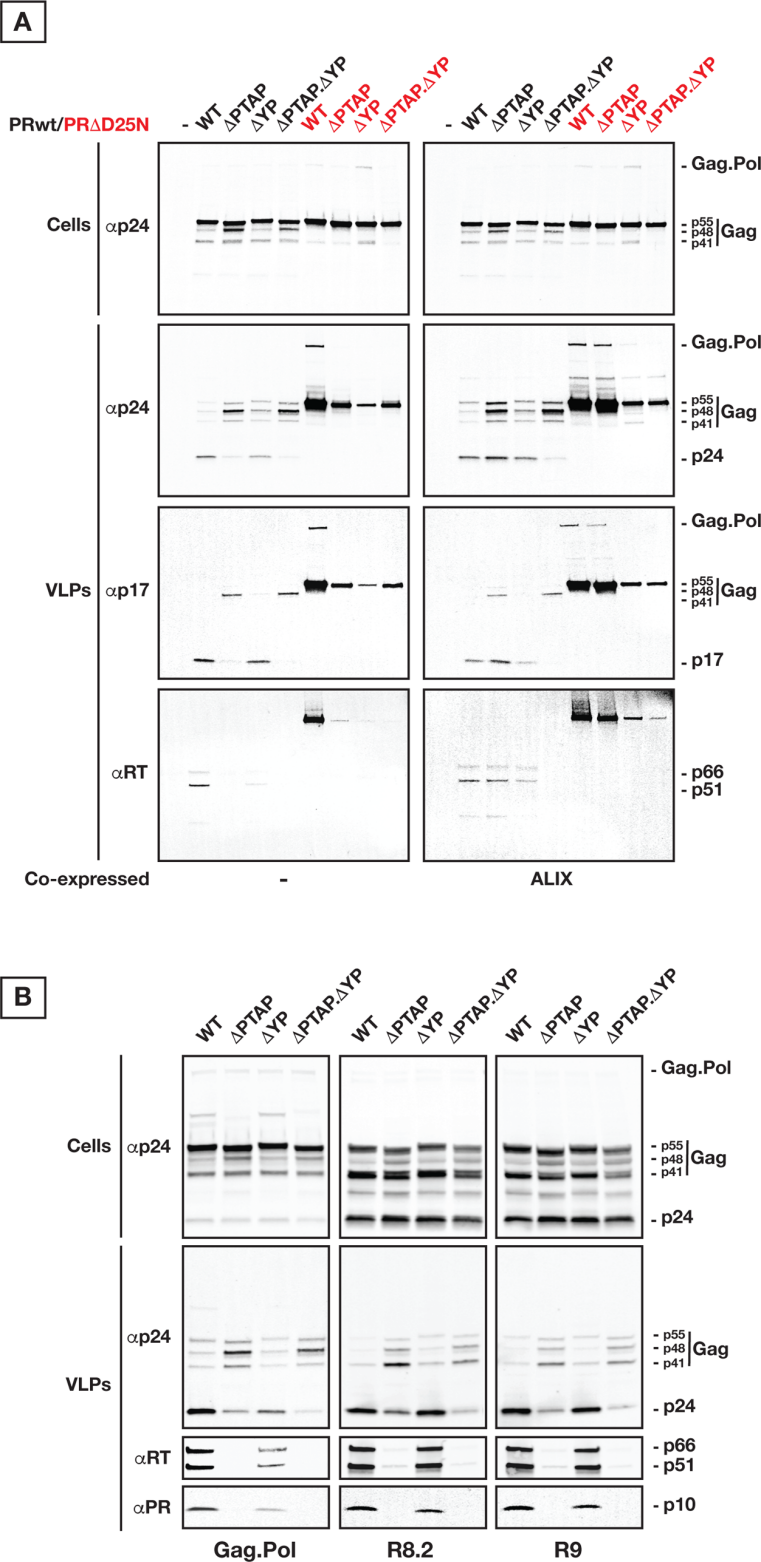
Gag p6 alteration induces the loss of Pol products due to delayed VLP release. **(A)** 250 ng of each Gag.Pol variants as indicated were used for transfection. Cells and VLPs were collected 24 hours post-transfection. Both Cells and VLPs were immunoprobed as indicated, respectively. Gag.Pol variants as indicated, with either active PR (in black) or inactive PR (in red), show distinct maturation profiles. Levels of incorporated RT are shown by immunoprobing the released VLPs. As control, ALIX is used to rescue the Gag.Pol ΔPTAP mutant. **(B)** Transfections were done using 250 ng of each vector, and samples were collected 24 hours post-transfection. Both Cells and VLPs were analyzed as indicated, respectively. Similar to the minimal Gag.Pol vector, HIV_R9_ and HIV_R8.2_ are sensitive to Tsg101 involvement via the PTAP site however contrary to Gag.Pol they are almost insensitive to ALIX interaction via the YP site. All experiments were performed 5 times with similar results, except for the R9-related panels that were processed two times with identical outcome.

Having established that the HIV Gag VLPs with abrogated interactions with Tsg101 and ALIX are delayed in their release, we set out to investigate the discrepancy in budding of HIV versus Gag VLPs. Upon transfection in cells, the HIV_R9_ or HIV_R8.2_ express Gag along with Gag-Pol and all other HIV co-factors aside from ENV for HIV_R8.2_. We chose to generate a system that only express Gag and Gag-Pol proteins for more accurate comparison with Gag to investigate whether the observed differences between Gag and HIV VLPs can be sufficiently explained by the packaging of Gag-Pol. We constructed the Gag plus Gag-Pol open reading frames in a single encoding cassette using humanized Gag and preserving the HIV ribosomal slippage (**S4A Fig**); the Gag plus Gag-Pol VLPs produced are referred to as “Gag.Pol”. We further generated variants of Gag.Pol by mutating p6 as described for **Fig 1** (ΔPTAP, ΔYP, and ΔPTAP. ΔYP), with either active (PRwt) or inactive protease (PRΔD25N; [42]). As shown in **Fig 4A**, expression of these plasmids in absence of any HIV accessory protein, promoted the production of VLPs incorporating both Gag and Gag-Pol proteins, and Gag processing was only observed in VLPs with PRwt. PRwt VLPs release and mature similarly to HIV virions (**Fig 4B**, WT lanes). Gag.Pol with ΔPTAP and ΔYP mutations resulted in formation of VLPs with defects in terms of VLP yield and maturation (**Fig 4**). Interestingly, PRΔD25N VLPs showed dramatic release defect in all p6 mutants (**Fig 4A**). Over-expression of ALIX substantially rescued the maturation defect due to ΔPTAP mutation (**Fig 4A**), as commonly reported.

Immunoprobing for Gag and Pol domains indicates that ΔPTAP VLPs are devoid of any detectable RT, while an average of 70% RT loss is observed in ΔYP VLPs (**Fig 4A and 4B** and **S5 Fig**). The RT loss is reversed in ΔPTAP VLPs by over-expression of ALIX as shown in **Fig 4A**. Interestingly, we observed that while ΔPTAP mutation induces identical RT loss in both Gag.Pol and HIV VLPs, the RT loss induced by ΔYP mutation in Gag.Pol VLPs is not occurring in HIV_R9_ and HIV_R8.2_ (**Fig 4B**). These data suggest the potential engagement of an HIV effector(s) missing in the minimal Gag.Pol system, that is likely capable of supporting ALIX function in the context of ΔYP mutation.

There is a reduction in the amount of incorporated RT within Gag.Pol p6 mutants when compared to incorporated RT in WT Gag.Pol. We hypothesized that delayed VLP release in addition to activation of PR before closure of the VLP neck would result in Pol auto-processing and subsequent diffusion of Pol products back to the host cytosol. Indeed, PR was also lost equivalently to RT in Gag.Pol p6 mutants, and follows the same profile in HIV_R9_ and HIV_R8.2_ ΔPTAP variants (**Fig 4B**). Supporting the notion of a race between VLP neck closure and PR activation, we also found that WT Gag.Pol showed a ~25% RT loss when compared to ΔPR Gag.Pol (**S5C Fig**). Based on the yields of Gag.Pol VLP production (comparing both PRwt and PRΔD25N to Gag VLPs), we suspected a longer delay in release of Gag.Pol VLPs with altered p6 compared to Gag VLPs.

### Gag.Pol VLP release is substantially delayed when p6 is altered

**Fig 5:**
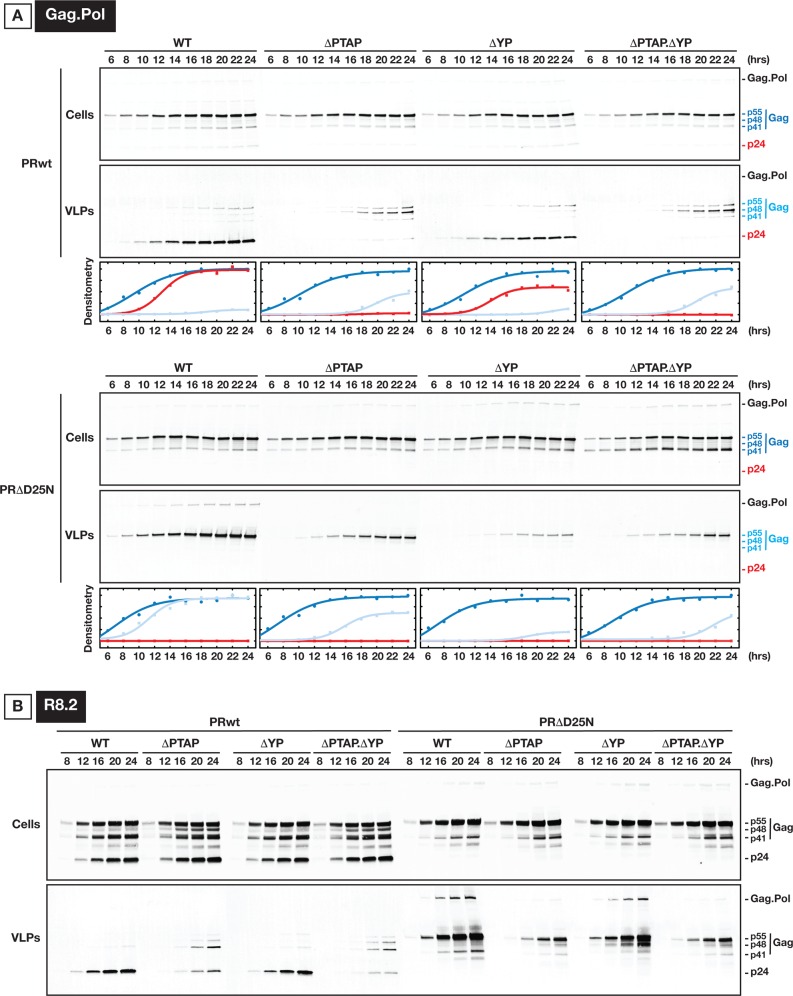
Kinetics of Gag.Pol processing and VLP release in U2OS cells. **(A)** The expression of Gag and Gag-Pol proteins are shown in cytosol along with their detection in released VLPs. 300 ng of each Gag.Pol construct were used for transfection. The top panels show Gag.Pol VLP production under WT as well as shown p6 mutations. The bottom panels show similar experiments using Gag.Pol PRΔD25N. The densitometry values plotted correspond to the band density on the immunoblotting. Gag and Gag-Pol were immunoprobed using p24 antibody. For accuracy, when Gag was partially processed, the quantified Gag p55 precursor is referred to the addition of p55, p48 and p41 bands. The color scheme: Gag p24 indicates full processing of the Gag and is shown in Red, the p41/48/55 is shown in dark blue to indicate cytosolic fraction and light blue to indicate the VLP fraction. **(B)** HIV VLP release is more sensitive to PTAP than to YP inactivation. 250 ng of each construct were used for transfection, and samples were collected at 4 hours intervals starting 8 hours post transfection. All experiments were performed 2 times with similar results.

VLP release kinetics of Gag.Pol variants were analyzed as shown in **Fig 5**. As expected, Gag.Pol VLPs budded out at a slower rate compared to Gag VLPs, likely due to the Pol cargo size. To this end, all delays related to p6 mutations were extended in time. Unlike Gag VLPs, which were released with a constant delay measured with respect to the cytosolic Gag concentration, the delay in Gag.Pol VLPs did not follow the same curve as the cytosolic fraction. These kinetics indicates the occurrence of parallel processes during Gag.Pol VLPs production.

Interestingly, in the context of PRwt (**Fig 5A, top panels**), we observed that the appearance of mature p24 versus p55 precursor and related products (p48 and p41) were not necessarily synchronized. Indeed, ΔYP mutation shows a delay in release of mature VLPs however their production does not continue to the same extent as for WT, instead, it saturates earlier while budding follows with VLPs enriched with Gag precursors. ΔPTAP mutation releases VLPs with mainly Gag precursors, especially Gag p48, and with a substantial delay. To test the effect of packaging full length Pol we performed kinetics on PRΔD25N (**Fig 5A, bottom panels**) with p6 mutations. VLP production kinetics in Gag.Pol PRΔD25N with p6 mutants were all significantly affected, strongly suggesting the importance of early ESCRT engagement (both Tsg101 and ALIX) when large cargo is loaded. Importantly, in any case, no full abrogation of VLP release was observed under any p6 mutation.

### Effect of Gag-cargo on VLP release

Our data show that p6 mutations create a delay in production of HIV Gag.Pol VLPs, which in turn results in premature activation of PR and diffusion of Pol components from budding VLPs. Also, the delay in VLP release was longer than the one measured for HIV Gag VLPs. To further dissect the mechanistic basis of the observed delay, we hypothesized that the delay length is associated with cargo size defined as domains added after HIV Gag protein, which are naturally present as Pol within HIV.

**Fig 6:**
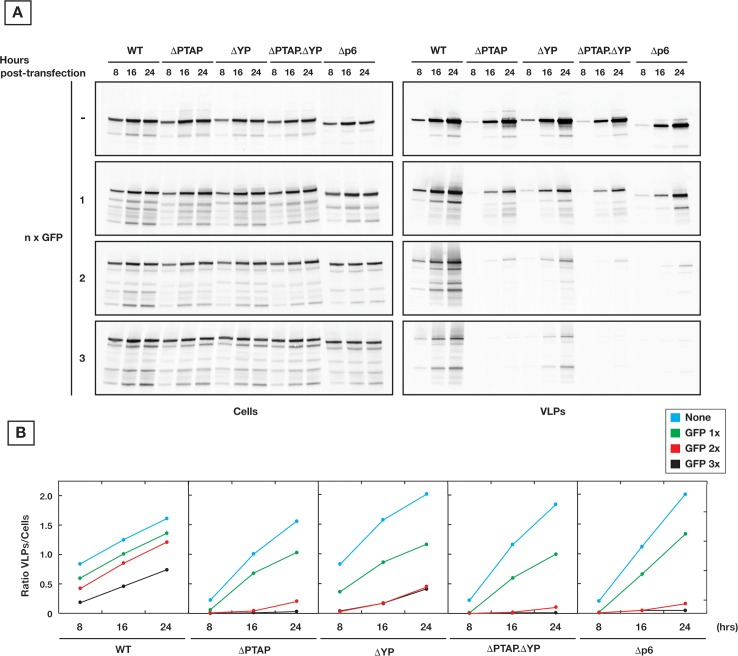
Gag cargo size dictates the requirement for p6. **(A)** The size of cargo fused to Gag was artificially modulated using tandem GFP proteins. Kinetics of VLP release in 293T cells of Gag with various number of tandem GFPs (n = 0, 1, 2 and 3) is shown. 200 ng of each Gag construct were used for transfection, and samples were collected at 8 hours intervals for 24 hours post-transfection. Release of Gag shows increased sensitivity to YP and PTAP mutations in presence of larger cargo sizes. **(B)** Densitometry values of the panels on (A) which correspond to the ratio of p24 in VLPs/Cells. All experiments were performed 3 times with similar results.

Our observations in budding kinetics of Gag.Pol VLPs demonstrated that when protease activation is inhibited and VLPs incorporate the full length Gag-Pol protein, the kinetics of VLP release is further delayed and becomes strongly dependent on early ESCRTs. These observations suggest a dependence of VLP release on cargo size. To evaluate the influence of cargo size on VLP production, we artificially fused GFPs in frame and in tandem to Gag C-terminus (in these experiments, every expressed Gag is in tandem with GFPs). We found that indeed VLP release by Gag-GFP_x_ variants is proportionally reduced depending on cargo length (x = 1, 2 or 3 GFPs). The p6 late domain mutation directly dictates the efficiency based on the severity of p6 alterations (**Fig 6**). These observations were confirmed by pulse/chase ^35^S-labeling experiments (**S2B Fig**).

**Fig 7:**
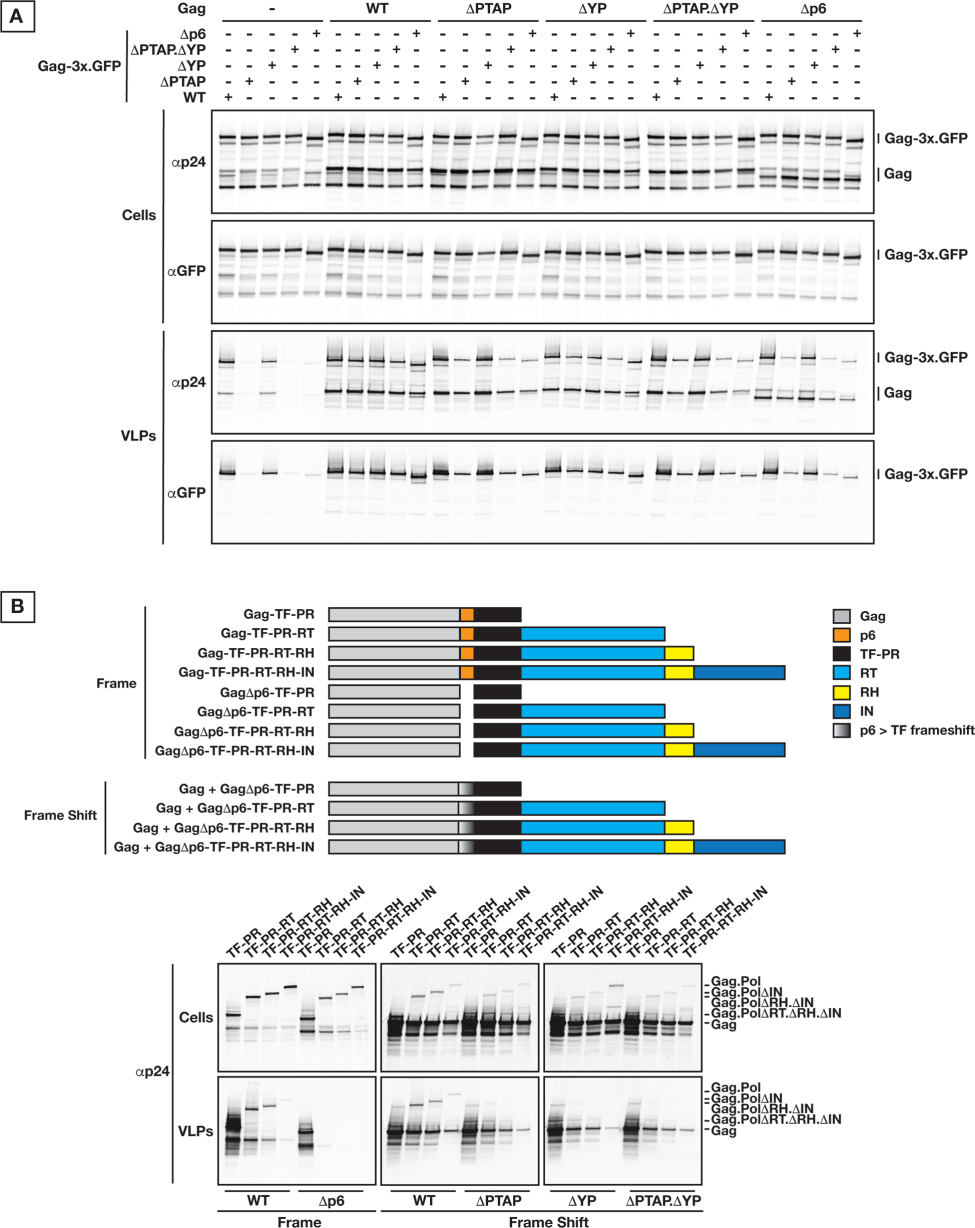
Gag cargo size is dependent on p6 for VLP release. **(A)** Rescue of VLP release from Gag-cargo with p6 mutations through expression of Gag p6 variants. 200 ng of each construct were used to transfect 293T cells; samples were collected 24 hours post-transfection. **(B)** Expression in 293T cells of natural cargo (Pol) truncations, depicted on the schematic representation (top panel), reproduced the same molecular phenotype as the artificial cargo (GFP) in terms of strict requirement of p6 for efficient VLP release (bottom panels). 250 ng of each Gag construct were used for transfection; samples were collected 24 hours post-transfection. All experiments were performed 3 times with similar results.

We further confirmed that intact Gag p6 is required for efficient VLP production with large cargo through rescue of p6 mutant Gag-3x.GFP VLP release by co-transfection of Gag with wt p6 (**Fig 7A**).There is a predominant impact for PTAP and at lower extent for YP.

We further modulated the cargo size using Pol truncations in the context of PRΔD25N for maintaining the integrity of Pol cargo. Experiments were performed both under physiological frame shifted expression of Gag.Pol along with Pol proteins expressed in frame with Gag which resulted in 10 fold increase of Pol incorporation in released VLPs. Under both conditions, we observed the same effect of p6 late domain mutations on VLP release (**Fig 7B**). In both cases, VLP production is negatively affected depending on the length of cargo and nature of p6 alteration.

In the context of truncated Gag.Pol with wild type protease, VLP production profile is more complex as deletions in Pol also influence timing of PR activation, as shown for Pol truncations (**S4C Fig**). The effect of p6 alteration on VLP release by Gag.Pol full length was similar to our findings above, however, in both cases of “in frame” or “frame shift” expression of Gag-Pol, PR activation appeared to be tightly regulated by Pol C-terminus, likely the IN domain. Indeed, when IN is deleted, PR was activated before VLP budding and this activation accounted for a substantial loss in VLP yield. This premature activation of Gag.Pol PRwt ΔIN (deletion of IN domain) seems likely to occur before Gag.Pol clusters on the plasma membrane as a ΔG2A mutation of the same Gag.Pol constructs showed exactly the same profile of PR premature processing. These findings are in line with the absence of VLP release when Gag-Pol full length is expressed in frame due to drastic delay of Gag-Pol VLP production (**S4B Fig**).

### Simulations of Gag.Pol VLP release

**Fig 8:**
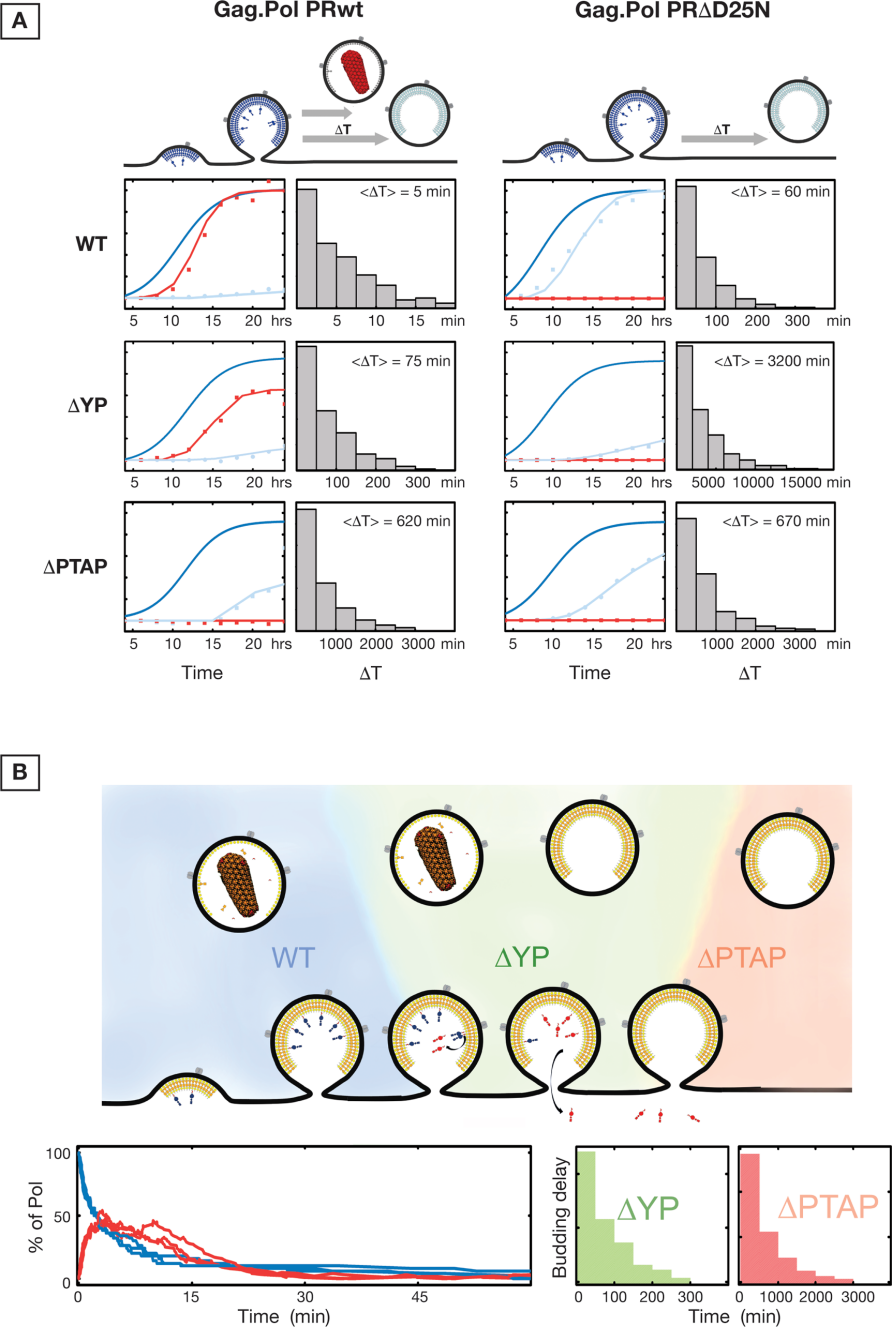
Simulation of Gag.Pol release kinetics. **(A)** Simulated curves (solid) fitting the experimental data (scatter) are shown for WT, ΔYP and ΔPTAP conditions of Gag.Pol PRwt and Gag.Pol PRΔ. Histograms show the distribution of delay times for each condition. **(B)** The number of Pol proteins attached to Gag (Blue) and cleaved but confined in the VLP (Red) are shown for three separate single VLP simulations.

Kinetics of Gag.Pol VLP release were analyzed using a Gellipsie stochastic algorithm [43] as detailed in Materials and Methods. This analysis incorporated a) the VLP release rates, b) protease activation kinetics, and c) diffusion of protease byproducts out of the open VLPs on the plasma membrane. The simulated data were fitted into the experimental data extracted from Gag.Pol VLP kinetics as shown in **Fig 8A**. Simulations allowed separation of the three underlying processes. As shown in **Fig 8A**, the delay in release of VLPs behaves along a poissonian curve with average delay times for WT, ΔYP and ΔPTAP alterations of 5 min, 75 min and 620 min, respectively. The delay in release of Gag.Pol PRΔD25N is substantially longer. During the simulations, the rates of protease activation and diffusion of protease byproducts were held constant while various p6 alterations were analyzed with varying VLP release rates; these rates are shown in **Fig 8B**. All together, the simulations support our hypothesis that a delay in release of VLPs, all other events constant, results in substantial loss of Pol associated enzymes from the VLPs.

## Discussion

Three major points emerge from our results: i) Late domain mutations of HIV Gag result in transient delay of virion release from the plasma membrane. ii) HIV protease is activated following full assembly of virions on the plasma membrane and delays in virion release result in loss of Pol associated enzymes to the cell cytosol and budding of non-infectious virions. iii) Size of cargo attached to the C-terminus of Gag modulates the speed and requirements for early ESCRT factors during HIV budding. While small cargo sizes rely mostly on Tsg101, larger cargo sizes are similarly dependent on both Tsg101 and ALIX for efficient VLP budding.

We show that alteration of Gag p6 late domains do not inhibit the release of HIV VLPs but rather result in delayed release. We characterized this effect for both VLPs that package HIV Gag only and for VLPs packaging both Gag and Gag-Pol (Gag.Pol). For the Gag.Pol VLPs, the delay ranges from ~70 minutes for the ΔYP mutants that lose proper interaction with ALIX to more than 10 hours for the ΔPTAP mutants which completely lose Tsg101 recruitment. Since the assembly of VLPs takes approximately 45 minutes, a ~10 fold delay in release of the budding VLP will result in a substantial accumulation of ΔPTAP VLPs at the cell surface when analyzed 12 to 24 hours post-transfection. ΔYP mutation has a much shorter delay of ~70 minutes and therefore would result in lesser fold increase in budding VLPs at the cell surface. Importantly, these accumulation levels of VLPs are consistent with the observed phenotypes of HIV late domain mutagenesis [5,27,15]. Interestingly, we observed that a pool of budding Gag.Pol and HIV VLPs undergo cellular endocytosis especially when release is slowed down due to p6 alteration; indeed, specifically inhibiting endocytosis substantially rescued the yield of VLP release by late domain mutants, especially for large cargo driven by Gag (Gag.Pol and HIV_R8.2_) (**S6 Fig**).

Our results rationally explain the infectivity assays previously reported on progeny virions lacking engagement of ESCRTs. Specifically, infectivity experiments using HIV_R8.2_ pseudotyped with VSV-G have shown that VLPs produced by HIV_R8.2_ ΔYP have a decreased infectivity of approximately 50% compared to wild type HIV_R8.2_ while HIV_R8.2_ ΔPTAP VLPs are non-infectious [44]. While these results could also indicate an alternate effect on particle release, a mismatch between released VLPs and their infectivity has been previously reported [45]. Analysis of the Gag.Pol VLP release kinetics suggests that activation of the protease is occurring immediately after completion of VLP assembly followed by Pol-associated enzymes diffusion out of VLPs in p6 mutants. The rates of PR activation and Pol product diffusion would result in the loss of all Pol enzymes ~60 minutes post-assembly as the VLPs remain open. Also, our analysis indicates that the VLP release times are distributed along a poissonian curve with an average of 5 minutes for WT, 70 minutes for ΔYP and 10 hours for ΔPTAP. This distribution of budding times correlates with percentage of Pol products lost in released ΔYP VLPs compared to WT VLPs. The ΔPTAP mutation which has a ~10 hours delay does not show Pol product incorporation.

HIV Gag protein alone is capable of budding from the plasma membrane. We found that Gag still efficiently buds out under severe p6 mutations but with delay at the cell surface for periods of ~20 minutes to ~1 hour. There is some minimal endocytosis of VLPs assembled under mutated Gag compared to Gag.Pol and HIV_R8.2_ VLPs, as shown in **S6 Fig**. The observed reduction of VLP release due to endocytosis is in agreement with a balance between fast budding and endocytosis of delayed VLPs. Prior to our observations it was shown that HIV Gag with mutated or even deleted p6 releases VLPs from cells [46–49]. These observations were interpreted as related to an ESCRT-independent release of Gag VLPs. In the context of HIV, the mismatch between the levels of VLP release and infectivity was also investigated as an indication of ESCRT-independent budding process and/or budding through intracellular vesicles and exocytosis [48]. Here, our data indicate that HIV virions defective in ESCRT recruitment mainly bud out from the plasma membrane but with proportional delays according to the severity of p6 late domain alterations. Aside from the mutations within the p6 domain, we have conducted extensive mutations within the NC domain of Gag. We found that in the context of Gag expression, VLP budding is independent of NC engagement with ALIX and/or indirectly Tsg101.

Interestingly, using a slight over-expression of CHMP4 and VPS4, we observed the incorporation of these proteins within released VLPs even in the context of severe p6 and NC mutations, and expression of VPS4DN markedly reduced the efficiency of VLP release. Engagement of Tsg101 and ALIX during the HIV budding is generally assumed to allow the recruitment of downstream ESCRT-III proteins which polymerize at the neck of the budding VLPs before release [15,50–54]. Based on our finding, we hypothesize direct recruitment of ESCRT-III and VPS4 to the neck of budding VLPs defective in early ESCRT engagement. To this end, we believe that, if this hypothesis is correct, the neck diameter formed in budding Gag VLPs is small enough to allow effective Tsg101/ALIX-independent CHMP recruitment and VLP release. *In vitro*, direct recruitment of CHMPs onto negatively curved membranes has been recently observed [55]. We cannot however rule out the possibility that ESCRT-III and VPS4 would be recruited in a Tsg101/ALIX-independent mechanism, possibly through engagement with AMOT and Nedd4 ubiquitin ligases [55–60]. In case of Gag.Pol and HIV VLP production, the Tsg101/ALIX-independent effective CHMP recruitment is substantially delayed due to the large cargo (Pol). We hypothesize that incorporation of Gag-Pol results in wider neck diameters. This hypothesis can rationally explain the different VLP release delays with altered p6 accordingly. The timing of recruitment of ALIX into HIV and EIAV has been investigated using Gag VLPs [61,62], based on our results we suggest that the recruitment may also be sensitive to cargo and therefore the recruitment should be further investigated in HIV virions incorporating both Gag and Gag-Pol. Finally, it is also possible that Tsg101/ALIX-independent CHMP recruitment to the neck of budding VLPs is naturally occurring, however, when Tsg101 and/or ALIX are involved during the CHMP recruitment, the process is faster and functions at maximum velocity to promote fast VLP release which promotes infectivity.

In line with the above, we found that ESCRT engagement during VLP budding grows more critical by addition of cargo to the Gag C-terminus. We have measured the kinetics of release for Gag.Pol VLPs with inactivated protease. The Pol protein has a large protein mass (150 kDa) and, in absence of processing, the full length Pol incorporates within the VLP. We found that under these conditions, Gag.Pol VLP production is similarly sensitive to Tsg101 as well as ALIX interactions as shown with ΔPTAP and ΔYP p6 mutants. These results are surprising since typically Tsg101 is the primary interaction during HIV VLP budding, however they agree with the increased importance of ALIX when the budding neck diameter is large like during cytokinesis [17,19,63]. Also, while Gag VLPs can still release efficiently even in the absence of functional late domains, addition of artificial cargos (GFPs in tandem) at the Gag C-terminus inhibits the VLP release in a cargo length-dependent manner. While these Gag-GFP experiments demonstrate the concept of cargo dependent ESCRT requirements, it does not directly reflect on effect of Pol in HIV-1 budding since the Gag-Pol comprises only 5–10% of Gags in the forming HIV virion. The rescue experiments with co-expression of Gag and GagΔp6-3xGFP are the closest comparison to the role of Pol in HIV budding. These experiments demonstrate efficient release of GagΔp6-3xGFP VLPs only when co-expressed with Gag which has a functional late domain. All these observations together support the mechanistic role of ESCRTs in accelerating the closure of budding VLPs with large necks, and that cargo size is the primary regulatory factor that dictates the early ESCRT requirement level.

Compared to the minimal Gag.Pol system, when HIV_R8.2_ PRΔD25N VLP release is tested (**Fig 5**), full length Pol (large cargo) release is more sensitive to PTAP integrity than to YP. We hypothesize that there is an HIV factor that is absent in the Gag.Pol and is promoting the efficient VLP release in absence of functional PTAP/YP sites. This factor is likely acting to mimic some of the Tsg101/ALIX function in accelerating ESCRT-III recruitment and/or promoting Pol packaging before PR activation.

The activation of HIV protease immediately post-assembly on plasma membrane is supported by some experimental evidence suggesting that increased packaging of Gag-Pol results in premature activation of PR [64]. Rapid maturation of HIV VLPs within 1 minute post-release has also been reported [65], although our results predict at least 30 minutes delay between release and full maturation. Also, processing was shown to be essential for HIV VLP release [60], however the rate of HIV assembly is not affected by PR inactivation [66].

**Fig 9:**
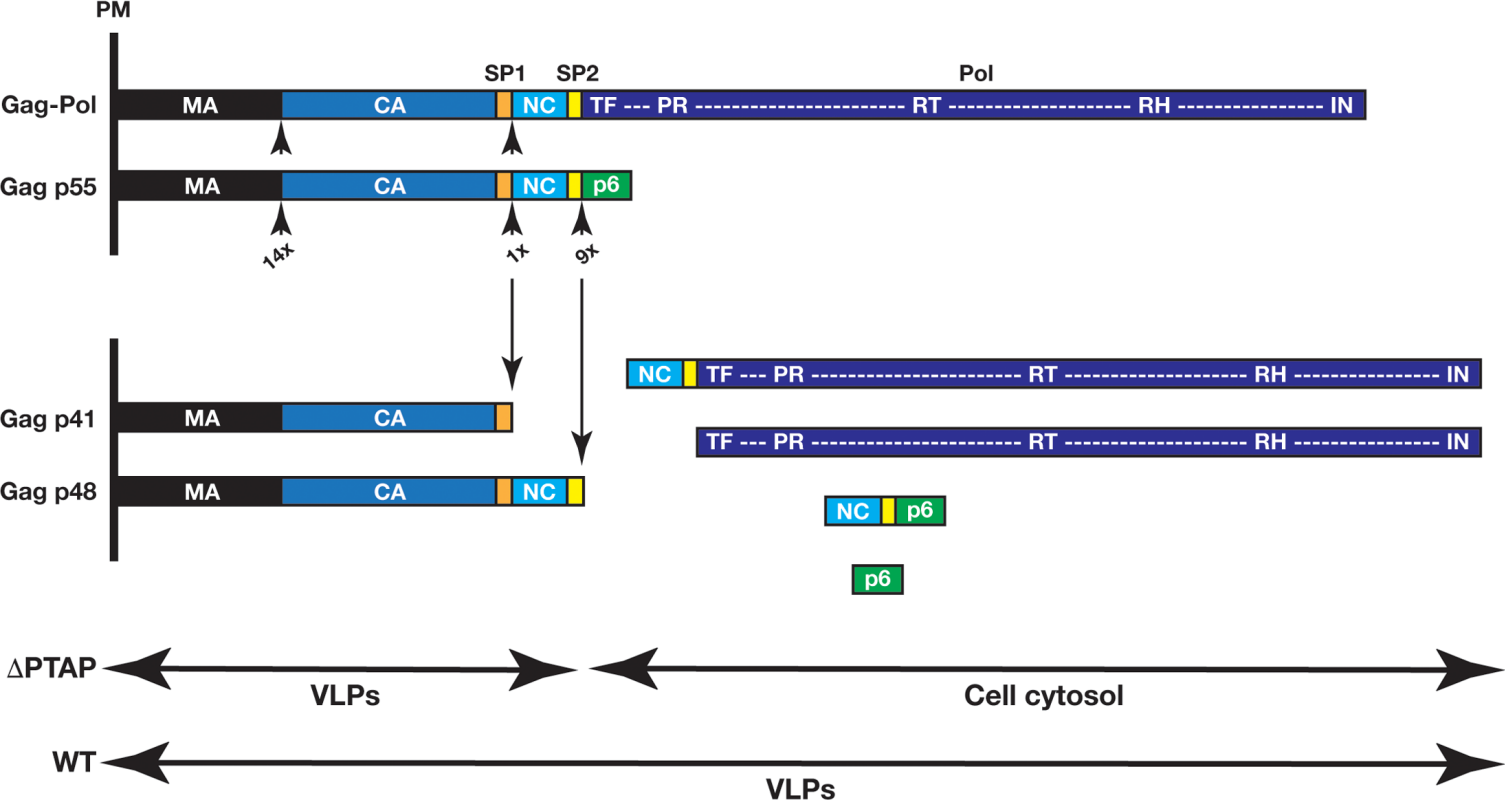
Products of Gag.Pol processing by PR during VLP production. Among cleavage sites in Gag and Gag-Pol, the SP1/NC and SP2/p6 sites are the most rapidly cleaved by PR [67]. If the neck closes under normal conditions (WT p6), soluble PR-containing fragments are trapped in VLPs and continue processing the remaining cleavage sites on Gag and Gag-Pol, which leads to release of mature virions. In the case of delayed neck closure, soluble PR-containing fragments diffuse to host cytosol and progeny virions are devoid of Pol products (ΔPTAP and to lesser extent ΔYP VLPs). PM, plasma membrane.

The observed kinetics of Gag precursor release from budding virions analyzed using computer simulations support activation of PR immediately post-virion assembly. Early biochemical characterization of PR cleavage sites showed that Gag and Gag-Pol SP1/NC and SP2/p6 sites are the first to get cleaved by PR [67]. Therefore, if the VLP neck closes before PR activation (as for WT p6 VLPs), soluble PR-containing fragments are trapped within the VLP and continue processing which results in virion maturation. In the case of delayed neck closure, soluble PR-containing fragments diffuse to host cytosol and the progeny virions produced lose Pol products based on the severity of p6 alteration. In agreement with our model (**Fig 9**), ΔPTAP and to lesser extent ΔYP VLPs are enriched mainly of Gag p48 and p41 forms, accordingly, clearly suggesting a loss of PR activity in these released VLPs.

The first report identifying the importance of the PTAP sequence within Gag p6 used RT activity within the released HIV virions as a measure of viral fitness [6]. In these pioneering experiments, HIV ΔPTAP virions lost RT activity, however, inactivation of PR restored RT activity within released HIV ΔPTAP virions. Our data explain this observation as shown in **Fig 4** and demonstrates that this phenotype is due to delayed release of ΔPTAP PRΔ VLPs with intact Pol domains.

All together, our observations suggest that the engagement of early ESCRTs during HIV budding is obligatory for speeding up the closure of budding virions and release of fully formed particles before the HIV protease activation occurs, which is fundamental for safeguarding the infectivity of progeny HIV virions. Other viruses and cellular processes whose cargo are not as time sensitive may forego some interactions with ESCRTs therefore possibly explaining the diverse requirements of ESCRTs in these processes [68,69].

Our observations show that ‘budding delay’ is a potent mechanism for inhibition of infectious retroviral release and suggest that this mechanism can be used for developing antiviral treatments that would not block ESCRT-dependent cellular processes but slow them to the point of infectious retroviral release inhibition. We also speculate that such mechanism maybe exploited by host cells to inhibit spread of infection.

## Materials and Methods

### Expression vectors, cells, and antibodies

HIV-1ΔR8.2 (HIV-1NL4-3 R9ΔApa [70]) and HIV R9 were used. Its late domain mutants, ΔPTAP and ΔYP were previously described [43]. Humanized Gag was produced as previously described [71]. ALIX (NM_013374), Tsg101 (NM_006292), CHMP4b (NM_176812) and VPS4A (NM_013245) were kindly provided by Dr. Wesley Sundquist (university of Utah) and were all HA N-terminally tagged. GFP ORF was cloned from peGFP-N1 (Clontech). Point mutations were introduced using the Quick Change site directed mutagenesis kit (Stratagene).

All cell lines used were grown in complete DMEM medium under standard conditions, excepted for TIRF experiments where cells were incubated in CO2-independent medium (LifeTechnologies).

Anti-ALIX [44], anti-Tsg101 (C-2, Santa Cruz Biotech.), anti-HA (HA.11 clone 16B12, Covance), anti-p24 (183-H12-5C, NIH AIDS Reagent Program), anti-p17 (17–1, Santa Cruz Biotech.), anti-RT (MAb21, NIH AIDS Reagent Program), anti-PR (1696, Santa Cruz Biotech.), and infrared dye coupled secondary antibodies (LI-COR) were used for immunoprobing. Scanning was performed with the Odyssey infrared imaging system (LI-COR) in accordance with the manufacturer’s instructions at 700 or 800 nm, accordingly.

### VLP release analysis

All cell lines used were transfected using lipofectamine 2000 (LifeTechnologies), except for 293T cells using standard CaPO4 precipitation technique. Both cells and media were collected for analysis. Cells were lysed in RIPA buffer (140 mM NaCl, 8 mM Na2HPO4, 2 mM NaH2PO4, 1% NP-40, 0.5% sodium deoxycholate, 0.05% SDS), and after removal of residual cell debris by centrifugation, VLPs were pelleted from cell supernatants by centrifugation for 2 hours through 10% (w/v) sucrose cushion at 15,000 x g. Final VLP samples were re-suspended in PBS. VLP release yields/ratio were calculated as VLPs-associated Gag forms per cell-associated Gag forms based on either CA or MA probing, after densitometry analysis of the immunoblotting data using the Image Studio Lite software (LI-COR). HIV Gag kinetics were fit using a boltzman equation to calculate the delay times for various mutants as described in Supporting Information.

### TIR-FM assessments

Live images were acquired using iMIC Digital Microscope made by TILL photonics controlled by TILL’s Live Acquisition imaging software (see also Supporting Information). U2OS cells were transfected with Gag-mCherry variants and observed by TIRF imaging. At 12 hours post-transfection, cells were gently detached using TryplE (LifeTechnologies). Detachment was achieved by removing the medium and washing once with PBS; a thin layer of TryplE was added to cover cells to allow cell to detach. Images of cells before detachment and afterwards with released VLPs left on the glass support are shown in **Fig 3** (right panels).

### MonteCarlo simulations

Simulations were setup following the Gillipsie algorithm [43]. Processing, diffusion of Pol and budding were simulated for a single VLP and repeated 500 times to generate a population. The expected p24 and p55 proteins were calculated based on the simulated VLP release. Three essential reactions were considered within each VLP as follows:

$$\frac{\delta [Gag.Pol]}{\delta t}=-k_{p}[Gag.Pol]\;*\;[Gag.Pol]$$

$$\frac{\delta [Pol]}{\delta t}=+k_{p}[Gag.Pol]\;*\;[Gag.Pol]-k_{d}[Pol]$$

$$\frac{\delta [VLP]}{\delta t}=-k_{r}[VLP]-k_{r}^{*}[{VLP}^{*}]$$

The concentration shown in brackets is the number of molecules within one VLP. At time t = 0 therefore $[Gag.Pol](t=0)=120\;(\frac{molecules}{VLP})\;and\;[Pol](t=0)=0$. In these equations *k*_*p*_ is the processing rate, *k*_*d*_ is the diffusion rate of Pol from the formed VLP with open neck, *k*_*r*_ is the rate of VLP release before processing, and $k_{r}^{*}$ is the rate of release after processing. The concentrations of p24 and p55 were calculated based on the following equations:

if([Gag.Pol]+[Pol]<2)then[p24]=0and[p55]=[Gag]+[Gag.pol]

if([Gag.Pol]+[Pol]>2)then[p24]=[Gag]+[Gag.Pol]and[p55]=0

Simulated curves of p24 and p55 (for this analysis, we did not distinguish between p41, p48 and p55, summing all products and representing them as p55) are used in **Fig 8A** to fit the experimental p24 and p55 concentrations measured in Gag.Pol kinetics experiments. In these simulations, *k*_*p*_ and *k*_*d*_ rates are kept constant while each specific p6 mutation is simulated with a corresponding *k*_*r*_. The simulated internal Pol (Red) and Gag.Pol (Blue) concentrations in three VLPs are shown in **Fig 8B**.

## Supporting Information

S1 Fig Characterization of the yield of VLP release by modulating Gag and its expression in 293T cells. **(A)** Effect of the G2A mutation. 200 ng of each Gag construct were used for transfection, and samples were collected 24 hours post-transfection. **(B)** Time course (0, 6, 12, and 24 hours) of VLPs release. 200 ng of each Gag construct were used for transfection. **(C)** Gag dose-dependent of VLPs release yields. 50, 100, and 200 ng of each Gag construct were used for transfection, and samples were collected 24 hours post-transfection. All panels correspond to Gag immunoprobing using p24 antibody. Experiments were performed 1 time for (A) and 3 times for (B) and (C) with very similar results.
(TIF)

S2 Fig Characterization of the yield of VLP release by Pulse/chase S35 labeling. **(A)** 200 and 250 ng of each Gag and HIV_R8.2_ constructs, respectively, were used for transfection. 11 hours post-transfection, cells were pulsed 1 hour then chased for 12 hours. Samples were collected and analyzed accordingly (see S1 Text). **(B)** Time course of VLPs release by Gag-nxGFP cargo. 200 ng of each Gag construct were used for transfection. Each 8 hours during the kinetic, cells were pulsed 30 min then chased the remaining time before samples collection. Both experiments in (A) and (B) were performed twice with the same outcome.
(TIF)

S3 Fig p6-independent VLP release by Gag is not cell type specific. Cells and VLPs were collected 24 hours post-transfection. All panels correspond to p24 immunoprobing. This experiment was performed 3 times with similar results.
(TIF)

S4 Fig Design and validation of the Gag.Pol vector developed for minimally mimicking HIV budding. **(A)** Comparison of the Gag-Pol vector previously used in the literature (Gan and Gould, 2012) and our Gag.Pol construct. (see S1 Text). 250 ng of each construct were used for transfection, and both Cells and VLPs were analyzed 24 hours post-transfection. **(B)** p6 re-introduction in the original GagPol vector (in frame) rescued VLP release, with reduced negative regulation of the integrase domain during the VLPs budding process. The original Gag-Pol vector consists of GagΔp6 fused in frame to Pol starting the TF domain till the IN (integrase) end. We fused full length Gag (p6 included) to Pol as in the original Gag-Pol vector and analyzed the VLPs release profile. 250 ng of each construct were used for transfection, and both Cells and VLPs were analyzed 24 hours post-transfection. **(C)** Full length Pol regulates proper PR activation during budding and release independently of intact Gag p6. Variants of our Gag.Pol p6 mutants were generated by modulating Pol length via Pol truncation as indicated. 250 ng of each construct were used for transfection, and both Cells and VLPs were analyzed 24 hours post-transfection. The vectors were expressed in 293T cells, and all panels correspond to p24 immunoprobing. All these experiments were performed at least 3 times with similar results.
(TIF)

S5 Fig Evaluation of RT yields in the VLPs released by Gag.Pol. **(A)** Independent triplicate assessments of VLPs release by the Gag.Pol variants in 293T cells as indicated, with either active PR (PRwt) or inactive PR (PRΔD25N). **(B)** Comparative evaluation of p24-related Gag products and RT in VLPs released by Gag.Pol PRwt versus PRΔ in 293T cells. Final VLP samples were re-suspended in the same volume, and the indicated volume folds were analyzed by immunoblotting as shown. **(C)** Densitometry values from Panels **(A)** and **(B)** were processed accordingly and plotted as the corresponding relative amounts compared to the standards that equalize to 1 for p24 in Gag.Pol PRwt and 100% for RT in Gag.Pol PRΔD25N. 250 ng of each construct were used for transfection, and VLPs were analyzed 24 hours post-transfection. These experiments were performed 3 times as shown for (A) with similar results.
(TIF)

S6 Fig Dynasore effect on VLP release. Dynasore, a noncompetitive inhibitor of the GTPase activity of Dynamin which blocks cellular dynamin-dependent endocytosis [72, 73], was used to assess VLP internalization during VLP release. Cells were treated with 80 μM Dynasore 4 hours post-transfection as previously described [73], and samples were collected 20 hours post-treatment. The vectors were expressed in 293T cells; all panels correspond to p24 immunoprobing. **(A)** Gag (200 ng of each construct were used for transfection). **(B)** Gag.Pol (250 ng of each construct were used for transfection). These experiments were performed 3 times with similar results.
(TIF)

S1 Text Supporting materials and methods. (DOCX)
