# Supplementary figures and images for: The Race against Protease Activation Defines the Role of ESCRTs in HIV Budding
Source: PLoS Pathog. 2016 Jun 9;12(6):e1005657. doi: 10.1371/journal.ppat.1005657 (PMC4900648; doi:10.1371/journal.ppat.1005657)

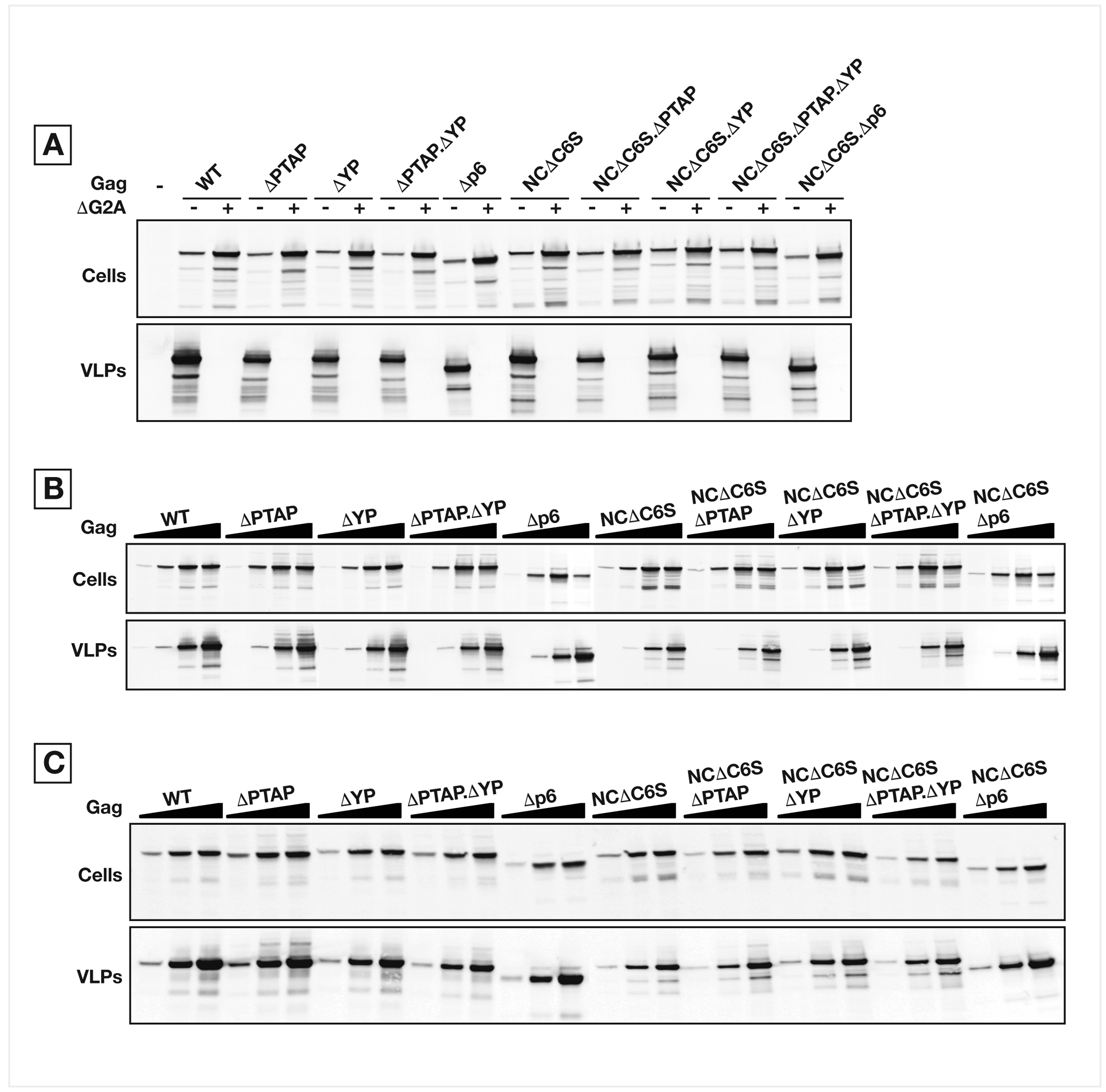

Supplement: S1 Fig — (A) Effect of the G2A mutation. 200 ng of each Gag construct were used for transfection, and samples were collected 24 hours post-transfection. (B) Time course (0, 6, 12, and 24 hours) of VLPs release. 200 ng of each Gag construct were used for transfection. (C) Gag dose-dependent of VLPs release yields. 50, 100, and 200 ng of each Gag construct were used for transfection, and samples were collected 24 hours post-transfection. All panels correspond to Gag immunoprobing using p24 antibody. Experiments were performed 1 time for (A) and 3 times for (B) and (C) with very similar results. (TIF) [file ppat.1005657.s001.tif]

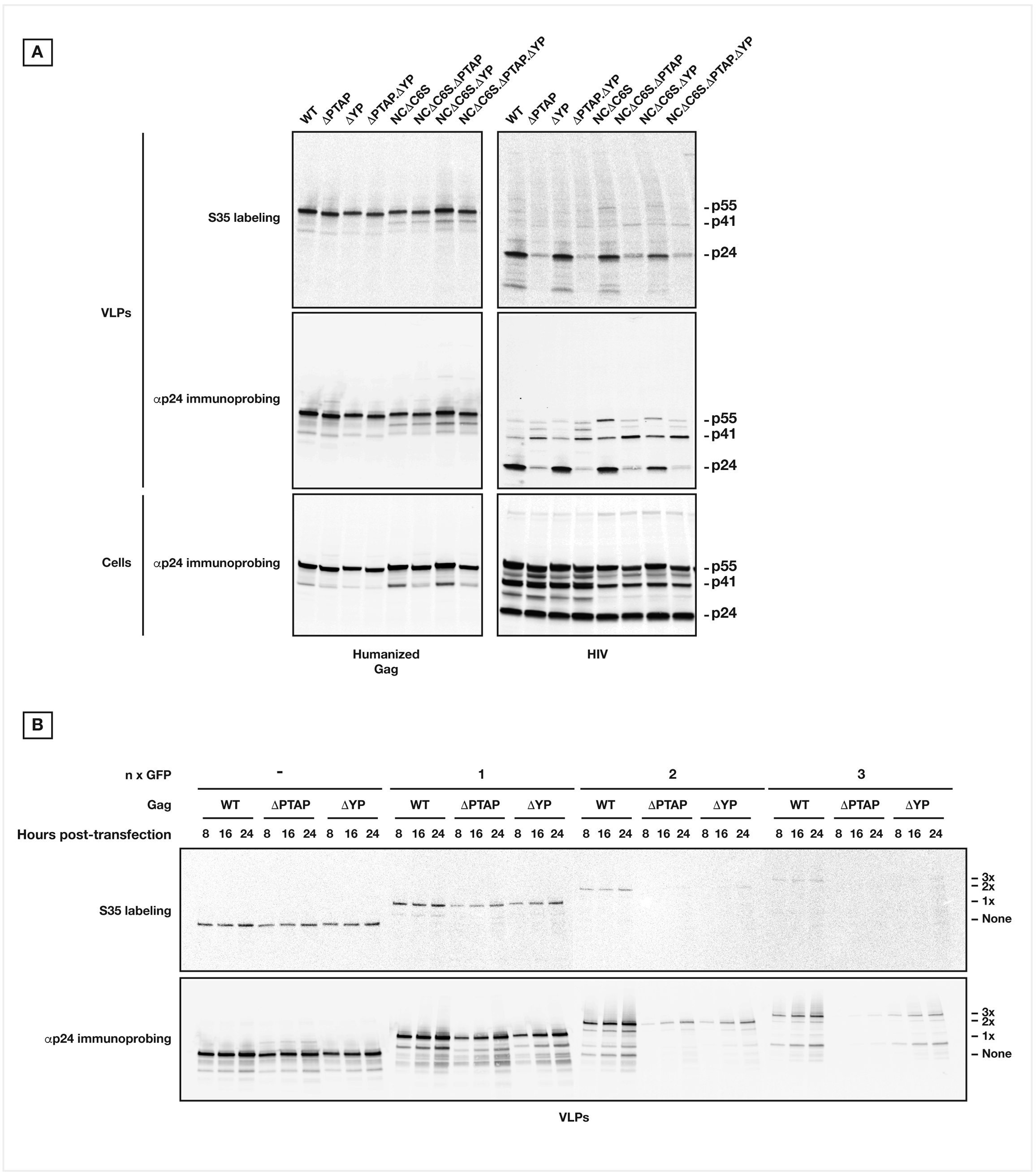

Supplement: S2 Fig — (A) 200 and 250 ng of each Gag and HIVR8.2 constructs, respectively, were used for transfection. 11 hours post-transfection, cells were pulsed 1 hour then chased for 12 hours. Samples were collected and analyzed accordingly (see S1 Text). (B) Time course of VLPs release by Gag-nxGFP cargo. 200 ng of each Gag construct were used for transfection. Each 8 hours during the kinetic, cells were pulsed 30 min then chased the remaining time before samples collection. Both experiments in (A) and (B) were performed twice with the same outcome. (TIF) [file ppat.1005657.s002.tif]

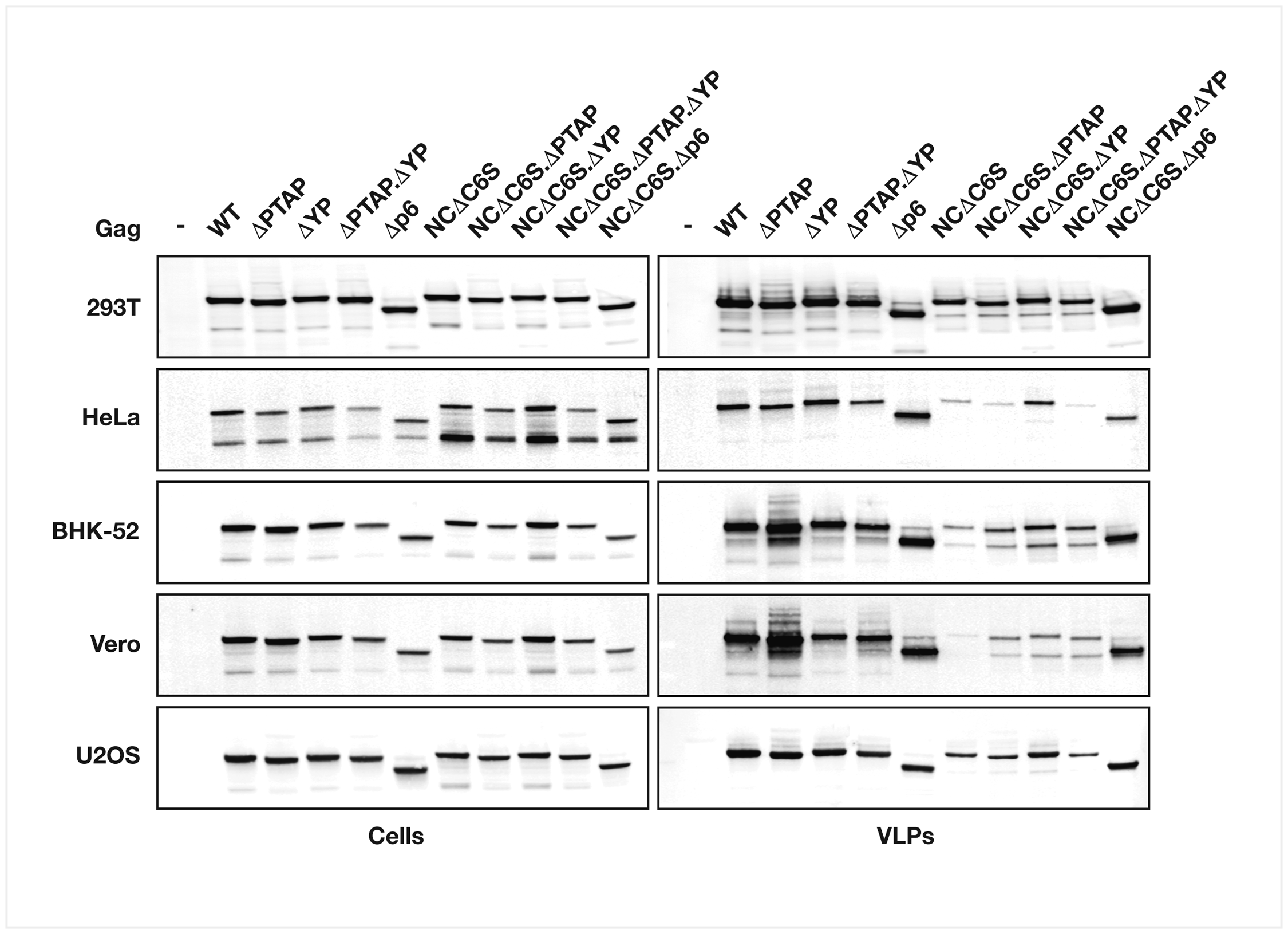

Supplement: S3 Fig — Cells and VLPs were collected 24 hours post-transfection. All panels correspond to p24 immunoprobing. This experiment was performed 3 times with similar results. (TIF) [file ppat.1005657.s003.tif]

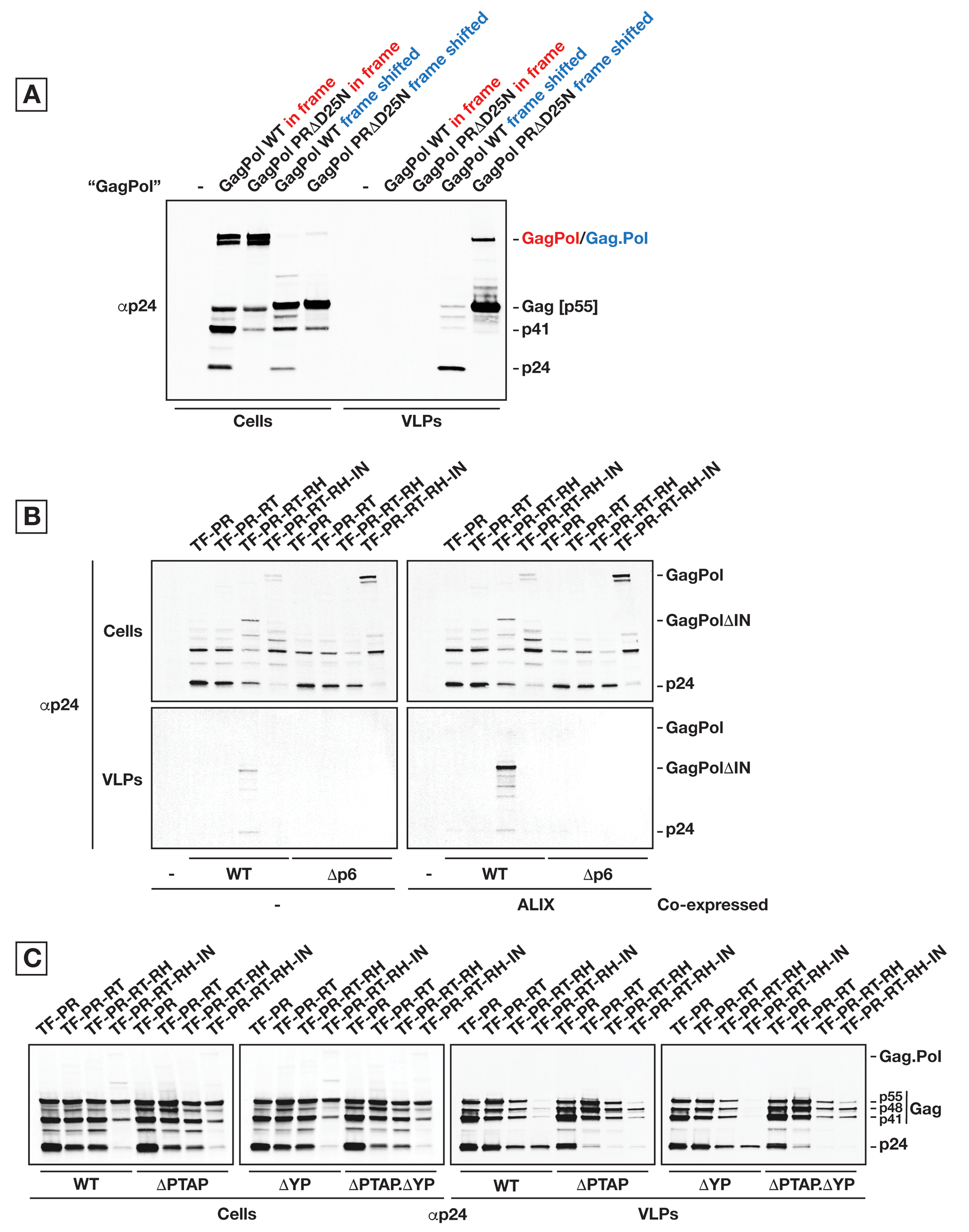

Supplement: S4 Fig — (A) Comparison of the Gag-Pol vector previously used in the literature (Gan and Gould, 2012) and our Gag.Pol construct. (see S1 Text). 250 ng of each construct were used for transfection, and both Cells and VLPs were analyzed 24 hours post-transfection. (B) p6 re-introduction in the original GagPol vector (in frame) rescued VLP release, with reduced negative regulation of the integrase domain during the VLPs budding process. The original Gag-Pol vector consists of GagΔp6 fused in frame to Pol starting the TF domain till the IN (integrase) end. We fused full length Gag (p6 included) to Pol as in the original Gag-Pol vector and analyzed the VLPs release profile. 250 ng of each construct were used for transfection, and both Cells and VLPs were analyzed 24 hours post-transfection. (C) Full length Pol regulates proper PR activation during budding and release independently of intact Gag p6. Variants of our Gag.Pol p6 mutants were generated by modulating Pol length via Pol truncation as indicated. 250 ng of each construct were used for transfection, and both Cells and VLPs were analyzed 24 hours post-transfection. The vectors were expressed in 293T cells, and all panels correspond to p24 immunoprobing. All these experiments were performed at least 3 times with similar results. (TIF) [file ppat.1005657.s004.tif]

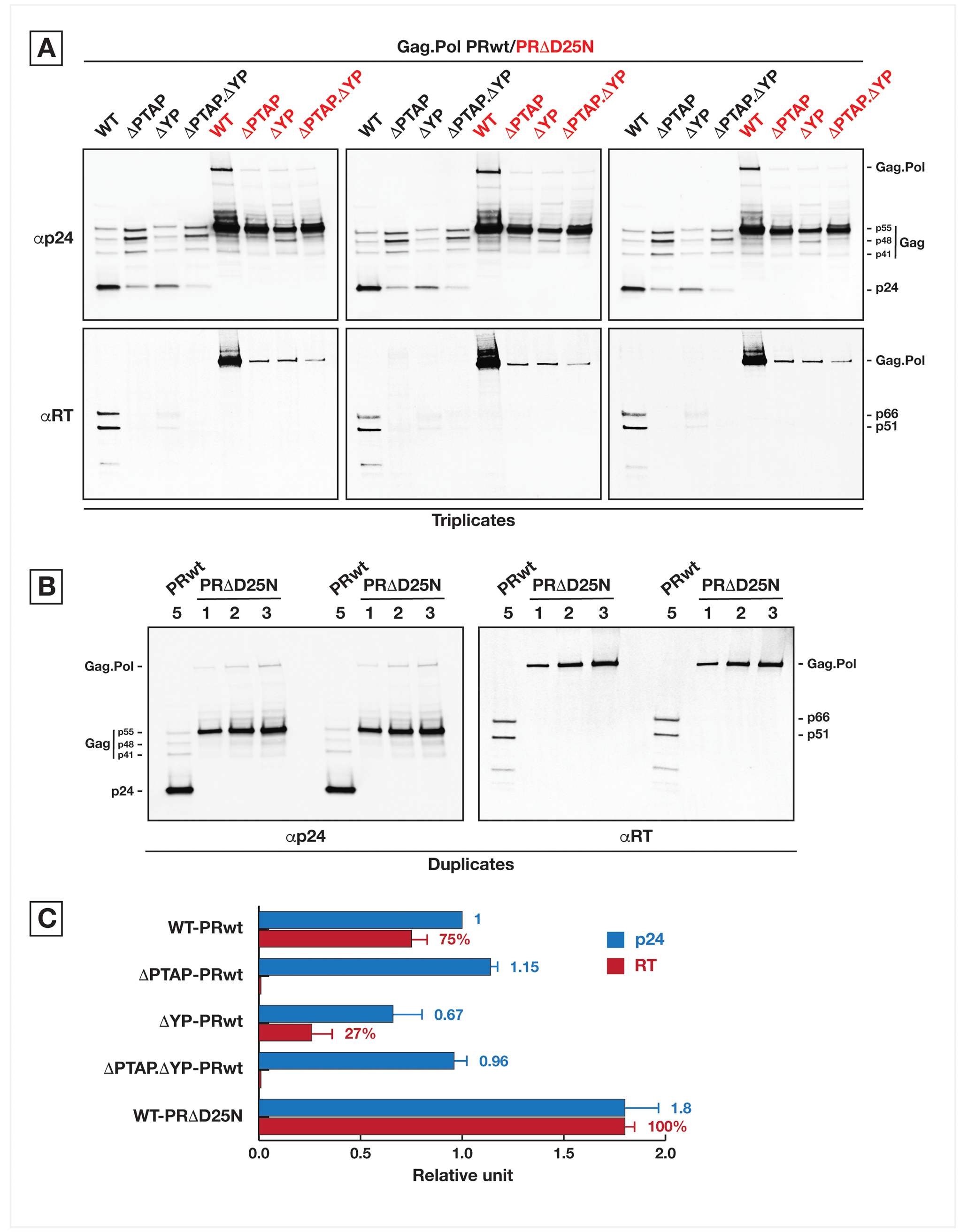

Supplement: S5 Fig — (A) Independent triplicate assessments of VLPs release by the Gag.Pol variants in 293T cells as indicated, with either active PR (PRwt) or inactive PR (PRΔD25N). (B) Comparative evaluation of p24-related Gag products and RT in VLPs released by Gag.Pol PRwt versus PRΔ in 293T cells. Final VLP samples were re-suspended in the same volume, and the indicated volume folds were analyzed by immunoblotting as shown. (C) Densitometry values from Panels (A) and (B) were processed accordingly and plotted as the corresponding relative amounts compared to the standards that equalize to 1 for p24 in Gag.Pol PRwt and 100% for RT in Gag.Pol PRΔD25N. 250 ng of each construct were used for transfection, and VLPs were analyzed 24 hours post-transfection. These experiments were performed 3 times as shown for (A) with similar results. (TIF) [file ppat.1005657.s005.tif]

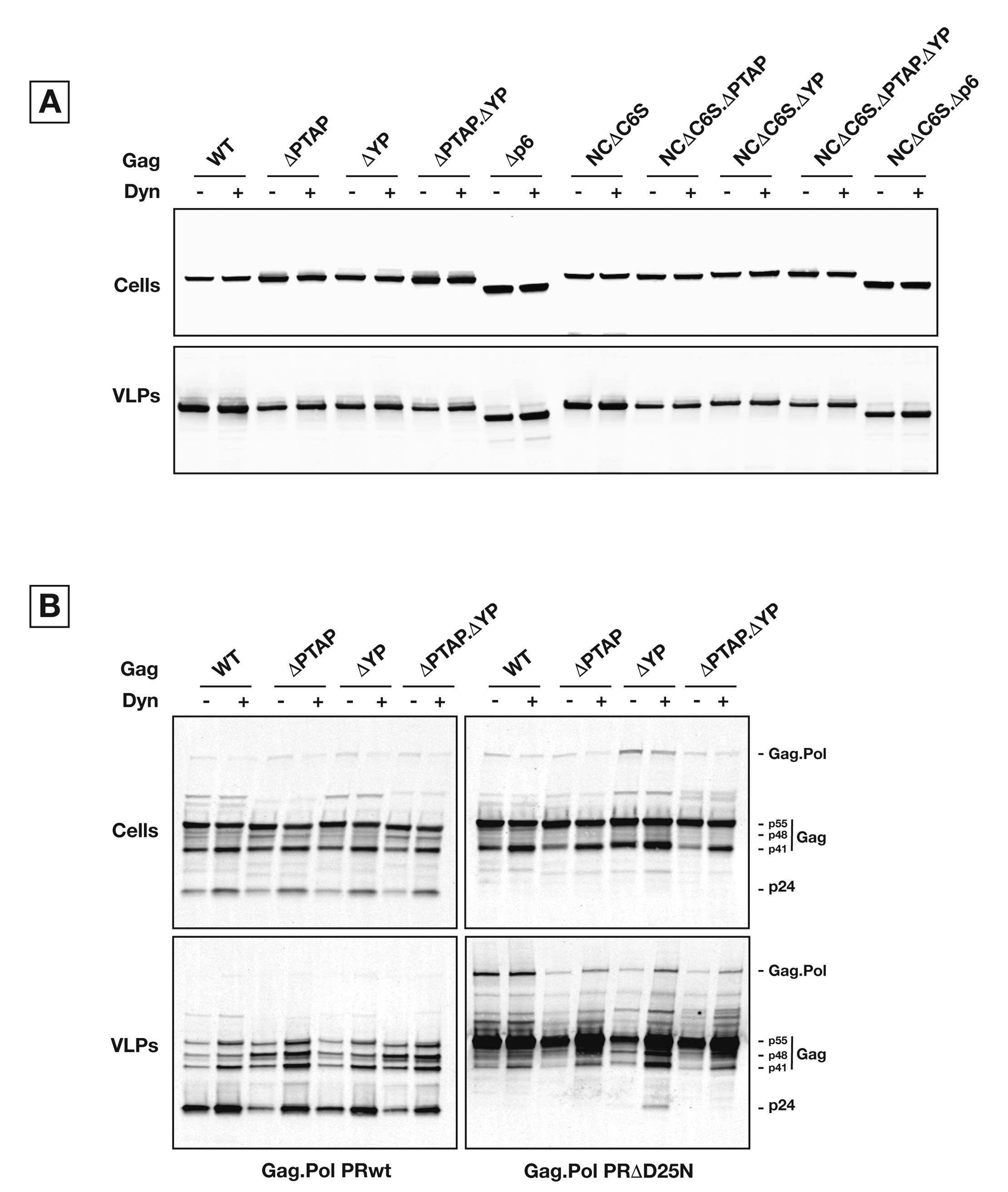

Supplement: S6 Fig — Dynasore, a noncompetitive inhibitor of the GTPase activity of Dynamin which blocks cellular dynamin-dependent endocytosis [72, 73], was used to assess VLP internalization during VLP release. Cells were treated with 80 μM Dynasore 4 hours post-transfection as previously described [73], and samples were collected 20 hours post-treatment. The vectors were expressed in 293T cells; all panels correspond to p24 immunoprobing. (A) Gag (200 ng of each construct were used for transfection). (B) Gag.Pol (250 ng of each construct were used for transfection). These experiments were performed 3 times with similar results. (TIF) [file ppat.1005657.s006.tif]
