# Supplementary material for: The Race against Protease Activation Defines the Role of ESCRTs in HIV Budding
Source: PLoS Pathog. 2016 Jun 9;12(6):e1005657. doi: 10.1371/journal.ppat.1005657 (PMC4900648; doi:10.1371/journal.ppat.1005657)
Supplement: S1 Text — (DOCX) [file ppat.1005657.s007.docx]

**SUPPORTING INFORMATION**

**The race against protease activation defines the role of ESCRTs in HIV budding**

Mourad Bendjennat and Saveez Saffarian

**Supporting Materials and Methods**

**Plasmids.**

***Humanized Gag ORF*.**

Gag ORF of HIV-1ΔR8.2 was humanized by codons optimization as previously described (Kofman et al., 2003) without altering the translated amino acid sequence. Natural (top) versus humanized (bottom) Gag nucleotidic sequences are shown below. Translated amino acid sequence corresponds to MA-CA-SP1-NC-SP2-p6. The NC cysteines (C), PTAP (x) and YP (x) are highlighted.

**ATG GGT GCG AGA GCG TCA GTA TTA AGC GGG GGA GAA TTA GAT CGA TGG GAA AAA ATT CGG TTA AGG CCA GGG GGA AAG**

**ATG GGC GCC CGC GCC TCC GTG CTG TCC GGC GGC GAG CTG GAC AGA TGG GAG AAG ATC CGC CTG CGC CCC GGC GGC AAG**

**M G A R A S V L S G G E L D R W E K I R L R P G G K**

**AAA AAA TAT AAA TTA AAA CAT ATA GTA TGG GCA AGC AGG GAG CTA GAA CGA TTC GCA GTT AAT CCT GGC CTG TTA GAA**

**AAG AAG TAC AAG CTG AAG CAC ATC GTG TGG GCC TCC CGC GAG CTG GAG CGC TTC GCC GTG AAC CCC GGC CTG CTG GAG**

**K K Y K L K H I V W A S R E L E R F A V N P G L L E**

**ACA TCA GAA GGC TGT AGA CAA ATA CTG GGA CAG CTA CAA CCA TCC CTT CAG ACA GGA TCA GAA GAA CTT AGA TCA TTA**

**ACC TCC GAG GGC TGC CGC CAG ATC CTG GGC CAG CTG CAG CCC TCC CTG CAA ACC GGC TCC GAG GAG CTG CGC TCC CTG**

**T S E G C R Q I L G Q L Q P S L Q T G S E E L R S L**

**TAT AAT ACA GTA GCA ACC CTC TAT TGT GTG CAT CAA AGG ATA GAG ATA AAA GAC ACC AAG GAA GCT TTA GAC AAG ATA**

**TAC AAC ACC GTC GCC ACG CTG TAC TGC GTG CAC CAG CGC ATC GAA ATC AAG GAC ACC AAG GAG GCC CTG GAC AAG ATC**

**Y N T V A T L Y C V H Q R I E I K D T K E A L D K I**

**GAG GAA GAG CAA AAC AAA AGT AAG AAA AAA GCA CAG CAA GCA GCA GCT GAC ACA GGA CAC AGC AAT CAG GTC AGC CAA**

**GAG GAG GAG CAG AAC AAG TCC AAG AAG AAG GCC CAG CAG GCC GCC GCC GAC ACC GGC CAT TCC AAC CAG GTG TCC CAG**

**E E E Q N K S K K K A Q Q A A A D T G H S N Q V S Q**

**AAT TAC CCT ATA GTG CAG AAC ATC CAG GGG CAA ATG GTA CAT CAG GCC ATA TCA CCT AGA ACT TTA AAT GCA TGG GTA**

**AAC TAC CCC ATC GTG CAG AAC ATC CAG GGC CAG ATG GTG CAC CAG GCC ATC TCC CCC CGC ACC CTG AAC GCC TGG GTG**

**N Y P I V Q N I Q G Q M V H Q A I S P R T L N A W V**

**AAA GTA GTA GAA GAG AAG GCT TTC AGC CCA GAA GTG ATA CCC ATG TTT TCA GCA TTA TCA GAA GGA GCC ACC CCA CAA**

**AAG GTG GTG GAG GAG AAG GCC TTC TCC CCC GAA GTC ATC CCC ATG TTC TCC GCC CTG TCC GAG GGC GCC ACC CCC CAG**

**K V V E E K A F S P E V I P M F S A L S E G A T P Q**

**GAT TTA AAC ACC ATG CTA AAC ACA GTG GGG GGA CAT CAA GCA GCC ATG CAA ATG TTA AAA GAG ACC ATC AAT GAG GAA**

**GAC CTG AAC ACC ATG CTG AAC ACC GTG GGC GGC CAC CAG GCC GCC ATG CAG ATG CTG AAG GAG ACC ATC AAC GAG GAG**

**D L N T M L N T V G G H Q A A M Q M L K E T I N E E**

**GCT GCA GAA TGG GAT AGA GTG CAT CCA GTG CAT GCA GGG CCT ATT GCA CCA GGC CAG ATG AGA GAA CCA AGG GGA AGT**

**GCC GCC GAG TGG GAC CGC GTG CAC CCC GTG CAC GCC GGC CCC ATC GCC CCC GGC CAG ATG CGC GAG CCC CGC GGC TCC**

**A A E W D R V H P V H A G P I A P G Q M R E P R G S**

**GAC ATA GCA GGA ACT ACT AGT ACT AGT ACC CTT CAG GAA CAA ATA GGA TGG ATG ACA CAT AAT CCA CCT ATC CCA GTA**

**GAC ATC GCC GGC ACC ACC TCC ACC AGT ACC CTG CAA GAG CAG ATC GGC TGG ATG ACC CAC AAC CCC CCC ATC CCC GTG**

**D I A G T T S T S T L Q E Q I G W M T H N P P I P V**

**GGA GAA ATC TAT AAA AGA TGG ATA ATC CTG GGA TTA AAT AAA ATA GTA AGA ATG TAT AGC CCT ACC AGC ATT CTG GAC**

**GGC GAG ATC TAC AAG CGC TGG ATC ATC CTG GGC CTG AAC AAG ATC GTG CGC ATG TAC TCC CCC ACC TCC ATC CTG GAC**

**G E I Y K R W I I L G L N K I V R M Y S P T S I L D**

**ATA AGA CAA GGA CCA AAG GAA CCC TTT AGA GAC TAT GTA GAC CGA TTC TAT AAA ACT CTA AGA GCC GAG CAA GCT TCA**

**ATC CGC CAG GGC CCC AAG GAG CCC TTC CGC GAC TAC GTG GAC CGC TTC TAC AAG ACC CTG CGC GCC GAG CAG GCC TCC**

**I R Q G P K E P F R D Y V D R F Y K T L R A E Q A S**

**CAA GAG GTA AAA AAT TGG ATG ACA GAA ACC TTG TTG GTC CAA AAT GCG AAC CCA GAT TGT AAG ACT ATT TTA AAA GCA**

**CAG GAG GTA AAG AAC TGG ATG ACC GAG ACC CTG CTG GTG CAG AAC GCC AAC CCC GAC TGC AAG ACC ATC CTG AAG GCC**

**Q E V K N W M T E T L L V Q N A N P D C K T I L K A**

**TTG GGA CCA GGA GCG ACA CTA GAA GAA ATG ATG ACA GCA TGT CAG GGA GTG GGG GGA CCC GGC CAT AAA GCA AGA GTT**

**CTG GGC CCC GGC GCC ACC CTG GAG GAG ATG ATG ACC GCC TGC CAG GGC GTG GGC GGC CCC GGC CAC AAG GCC CGC GTG**

**L G P G A T L E E M M T A C Q G V G G P G H K A R** **V**

**TTG GCT GAA GCA ATG AGC CAA GTA ACA AAT CCA GCT ACC ATA ATG ATA CAG AAA GGC AAT TTT AGG AAC CAA AGA AAG**

**CTG GCC GAG GCC ATG TCC CAA GTC ACC AAC CCC GCC ACC ATC ATG ATC CAG AAG GGC AAC TTC CGC AAC CAG CGC AAG**

**L A E A M S Q V T N P A** **T I M I Q K G N F R N Q R K**

**ACT GTT AAG TGT TTC AAT TGT GGC AAA GAA GGG CAC ATA GCC AAA AAT TGC AGG GCC CCT AGG AAA AAG GGC TGT TGG**

**ACC GTG AAG TGC TTC AAC TGC GGC AAG GAG GGC CAC ATC GCC AAG AAC TGC CGC GCC CCC CGC AAG AAG GGC TGC TGG**

**T V K C F N C G K E G H I A K N C R A P R K K G C W**

**AAA TGT GGA AAG GAA GGA CAC CAA ATG AAA GAT TGT ACT GAG AGA CAG GCT AAT TTT TTA GGG AAG ATC TGG CCT TCC**

**AAG TGC GGC AAG GAG GGC CAC CAG ATG AAA GAT TGT ACT GAG AGA CAG gct aat ttt tta ggg aag atc tgg cct tcc**

**K C G K E G H Q M K D C T E R Q A N F L G K I W P S**

**CAC AAG GGA AGG CCA GGG AAT TTT CTT CAG AGC AGA CCA GAG CCA ACA GCC CCA CCA GAA GAG AGC TTC AGG TTT GGG**

**cac aag gga agg cca ggg aat ttt ctt cag agc aga cca gag cca aca gcc cca cca gaa gag agc ttc agg ttt ggg**

**H K G R P G N F L Q S R P E P T A P P E E S F R F G**

**GAA GAG ACA ACA ACT CCC TCT CAG AAG CAG GAG CCG ATA GAC AAG GAA CTG TAT CCT TTA GCT TCC CTC AGA TCA CTC**

**gaa gag aca aca act ccc tct cag aag cag gag ccg ata gac aag gaa ctg tat cct tta gct tcc ctc aga tca ctc**

**E E T T T P S Q K Q E P I D K E L Y P L A S L R S L**

**TTT GGC AGC GAC CCC TCG TCA CAA TAA**

**ttt ggc agc gac ccc tcg tca caa taa**

**F G S D P S S Q ***

***Gag.Pol system*.**

The original Gag-Pol vector consists of GagΔp6 fused in frame to Pol starting the TF domain till the IN (integrase) end. Our Gag.Pol vector is strictly reproducing the HIV Gag-Pol nucleotidic sequence, which include the complete Gag ORF and the ribosomal frame shift TTTTT sequence that allow the Gag ORF, at the SP2 translation start, to shift and follow now with the start of Pol (TF segment) generating a larger Gag-Pol ORF (*** indicate the frame shift location).

**AAG TGC GGC AAG GAG GGC CAC CAG ATG AAA GAT TGT ACT GAG AGA CAG gct aat ttt tta ggg aag atc tgg cct tcc**

**K C G K E G H Q M K D C T E R Q A N F L G K I W P S**

**> SP2**

**AAG TGC GGC AAG GAG GGC CAC CAG ATG AAA GAT TGT ACT GAG AGA CAG gct aat ttttt agg gaa gat ctg gcc ttc**

**K C G K E G H Q M K D C T E R Q A N F R E D L A F**

**> TF *****

We used humanized Gag instead of the natural Gag to optimize expression protein level. The VLPs released correspond to a mix of Gag and Gag-Pol proteins under a ratio of 1/15 to 1/20 Gag-Pol/Gag. Importantly, the original Gag-Pol vector that does not express Gag and corresponds to 100% GagPolΔp6 was incapable of releasing VLPs.

**Densitometry.**

We used the Odyssey infrared imaging system (LI-COR) for scanning the immunoblots and processing the raw data and following the manufacturer’s instructions no signals were saturated. Where quantification of immunoblots were performed, western blotting was also performed on a 2X dilution series of the maximum signal within the experimental results. The band intensities were quantified using the LI-COR Image Studio Line software. To convert the intensities to relative concentrations, the linear range within the dilution series was used to calculate corrected intensity values. These corrected intensities were plotted against original intensities and a polynomial equation with cubic power was used to generate a model for corrected intensities. This cubic polynomial equation was then used to assign values to individual bands within the experiments therefore correcting for both the low intensity as well as high intensity non linearity within the western blots.

***Kinetics of Gag VLPs release.***

VLPs production by all Gag p6 variants was similar after 24 hours, we then investigated the kinetics of VLPs release by Gag WT, ΔPTAP, ΔYP and Δp6 along with ΔG2A in U2OS cells using western blotting and densitometry analysis of the immunoblotting data. Cells and VLPs were collected hourly and probed using p24 antibody as shown in **Figure 3**. The concentrations of all these Gag variants expressed in cells increases along a sigmoidal curve. We used the following equation to empirically capture their concentrations within the cytosol:

$\left[ Gag \right]=A\left[ 1-\frac{1}{1+e^{-\alpha\left( t-t_{0} \right)}} \right]$ **(1)**

In which α was experimentally determined based on fits to Gag’s concentrations in cytosol to be 0.87. The concentration of Gag was normalized to the maximum concentration of Gag WT in the cytosol after 12 hours. The parameter A captures any variations in expression between Gag mutants. We found that equation **(1)** also fits the Gag released in VLPs with identical α of 0.87 albeit different values for t_0_. Using equation **(1)** to fit the data collected, we estimated the values of t_0_ for each condition as following:

|  | t_0_^Cytosol^ (hours) | t_0_^VLPs^ (hours) | ΔT (Δt_mutant_ – Δt_WT_) (min) |
| --- | --- | --- | --- |
| WT | 8.7 ± 0.3 | 12.1 ± 0.1 | - |
| ΔPTAP | 8.5 ± 0.3 | 12.8 ± 0.1 | 55 ± 15 |
| ΔYP | 8.6 ± 0.3 | 12.3 ± 0.1 | 20 ± 15 |
| Δp6 | 8.7 ± 0.3 | 12.9 ± 0.1 | 50 ± 15 |
| ΔG2A | 8.1 ± 0.3 | $\infty$ | $\infty$ |

***Kinetics of Gag.Pol VLPs release.***

As shown in **Figure 5**, we measured the kinetics of VLPs release upon expression of the Gag.Pol variants in U2OS cells starting from 6 hours post-transfection to 24 hours. At two hours intervals, both cells and released VLPs were collected for analysis by western blotting. To test the effect of the late domain mutations on the kinetics and composition of the released VLPs, we tested WT, ΔPTAP, ΔYP, ΔPTAP+ΔYP mutations in the context of PRwt as well as inactive PRΔD25N. Western blots involved p24 antibody immunoprobing for visualizing both Gag and Gag-Pol precursors as well as processed p24-related products in the released VLPs. All kinetics were fitted to the experimental Boltzman curve described in Equation **(1)**. The concentration increase of Gag-related products within the cytosol showed gradual accumulation followed by fast increase and later saturation. The profile within the cytosol was consistent between all various mutants and therefore it was possible to fit all the cytosolic data with α value of 2.5. The cytosolic t_0_ times varied from 9 to12 hours as shown below.

|  | Cytosol p55 | | VLP p24 | | VLP p55 | |
| --- | --- | --- | --- | --- | --- | --- |
|  | t_0_ (hrs) | α | t_0_ (hrs) | α | t_0_ (hrs) | α |
| PRwt | 9.4 ± 0.4 | 2.5 | 12.9 ± 0.2 | 1.5 | 18.4 ± 0.4 | 1.5 |
| PRwt + ΔPTAP | 10.5 ± 0.4 | 2.5 | $\infty$ | 1.5 | 19.6 ± 0.4 | 1.5 |
| PRwt + ΔYP | 10.3 ± 0.4 | 2.5 | 14.0 ± 0.4 | 1.5 | 22.0 ± 0.3 | 1.5 |
| PRwt + ΔPTAP+ΔYP | 10.5 ± 0.4 | 2.5 | $\infty$ | 1.5 | 19.6 ± 0.3 | 1.5 |
| PRΔ | 6.9 ± 0.8 | 2.5 | − | − | 11.25 ± 0.4 | 1.5 |
| PRΔ + ΔPTAP | 7.3 ± 0.7 | 2.5 | − | − | 15.8 ± 0.3 | 1.5 |
| PRΔ + ΔYP | 7.0 ± 0.6 | 2.5 | − | − | 18.7 ± 0.1 | 1.5 |
| PRΔ + ΔPTAP + ΔYP | 9.0 ± 0.5 | 2.5 | − | − | 21.6 ± 0.8 | 1.5 |

**TIR-FM microscope.**

Live images were acquired using iMIC Digital Microscope made by TILL photonics controlled by TILL’s Live Acquisition imaging software. 561 nm diode-pumped solid state (DPSS) laser (Cobolt Jive, 561 nm Jive High Power) was used to excite mCherry. Laser beam passed through an AOTF (acousto-optical tunable filter) and focused into a fiber that delivers the light to TILL Yanus digital scan head and then Polytrope II optical mode switch. Yanus consists of two galvo-mirrors and one spherical mirror to control the laser beam position. The Polytrope rapidly switches illumination beam path between Epi (wide field), FRAP and TIRF microscopy modes. It also holds the quadrant photodiode used for TIRF penetration depth calibration, which was set to 180 nm for the experiments in this manuscript. In the TIRF mode Yanus is used to control the position of the focused beam in the objective’s back focal plane and can be adjusted within 0.2 milliseconds. The focused beam was positioned at the edge of the back focal plane of the objective (N = 1.46, 100X, Zeiss) to reach beyond the critical angle and achieve TIRF. TIRF critical angle was verified by scanning the laser beam across the back aperture and measuring the reflection of the laser from the Glass sample interface back into the objective and onto the quadrant photodiode. The penetration depth of the beam is calculated based on the incident angle of the beam that is in turn measured by the position of the beam on the quadrant photodiode. Once the penetration depths for the experiments are set at the beginning of acquisition, a feedback loop keeps the focus of the objective on the sample by constantly monitoring the position of the back-reflected beam with respect to the original beam. We also rotated the TIRF illumination on the objective back focal plane 1 turn/exposure (TIRF360) to maximize homogeneity of the TIRF images.

**References**

Gan X, Gould SJ. HIV Pol inhibits HIV budding and mediates the severe budding defect of Gag-Pol. Plos One. 2012; 7: e29421.

Kirchhausen T, Marcia E, Pelish HE. Use of dynasore, the small molecule inhibitor of Dynamin, in the regulation of endocytosis. Methods Enzymol. 2008; 438: 77-93.

[Kofman A](http://www.ncbi.nlm.nih.gov/pubmed/?term=Kofman%20A%5BAuthor%5D&cauthor=true&cauthor_uid=12683241), [Graf M](http://www.ncbi.nlm.nih.gov/pubmed/?term=Graf%20M%5BAuthor%5D&cauthor=true&cauthor_uid=12683241), [Bojak A](http://www.ncbi.nlm.nih.gov/pubmed/?term=Bojak%20A%5BAuthor%5D&cauthor=true&cauthor_uid=12683241), [Deml L](http://www.ncbi.nlm.nih.gov/pubmed/?term=Deml%20L%5BAuthor%5D&cauthor=true&cauthor_uid=12683241), [Bieler K](http://www.ncbi.nlm.nih.gov/pubmed/?term=Bieler%20K%5BAuthor%5D&cauthor=true&cauthor_uid=12683241), [Kharazova A](http://www.ncbi.nlm.nih.gov/pubmed/?term=Kharazova%20A%5BAuthor%5D&cauthor=true&cauthor_uid=12683241), [Wolf H](http://www.ncbi.nlm.nih.gov/pubmed/?term=Wolf%20H%5BAuthor%5D&cauthor=true&cauthor_uid=12683241), [Wagner R](http://www.ncbi.nlm.nih.gov/pubmed/?term=Wagner%20R%5BAuthor%5D&cauthor=true&cauthor_uid=12683241). HIV-1 gag expression is quantitatively dependent on the ratio of native and optimized codons. Tsitologiia. 2003; 45: 86-93.

Marcia E, Ehrlich M, Massol R, Boucrot E, Brunner C, Kirchhausen T. Dynasore, a cell-permeable inhibitor of Dynamin. Developmental Cell. 2006; 10: 839–850.
